# Boosting Bulk‐to‐Surface Electron Transfer in CeO_2_ via Oxygen Vacancy Channels for Ultrafast NO_2_ Sensing

**DOI:** 10.1002/advs.202523186

**Published:** 2026-01-20

**Authors:** Yucheng Ou, Fuwen Wang, Nana Xu, Haiyang Song, Tao Liu, Bing Wang, Ming Zhang, Lei Liao, Hui Xu, Haijun Liu, Qingjiang Li, Wei Wang

**Affiliations:** ^1^ College of Electronic Science and Technology National University of Defense Technology Changsha China; ^2^ Science and Technology on Advanced Ceramic Fiber and Composites Laboratory College of Aerospace Science and Engineering National University of Defense Technology Changsha China; ^3^ School of Materials Science and Engineering Hunan University of Science and Technology Xiangtan China; ^4^ Changsha Semiconductor Technology and Application Innovation Research Institute College of Semiconductors (College of Integrated Circuits) Hunan University Changsha China

**Keywords:** CeO_2_, gas sensor, NO_2_ detection, oxygen vacancies, response‐recovery time

## Abstract

Modulating the electron transport dynamics in gas sensors is crucial for achieving rapid‐response gas detection. However, polaron localization causes sluggish electron migration from the bulk to the surface, severely limiting surface electron concentration and reaction activity. In this work, we leverage the difference in migration barriers of surface V_o_ in CeO_2_ to drive the directional migration of surface V_o_ into the bulk via precisely controlled thermal treatment, thereby forming electron transfer channels bridging the bulk and the surface. Experiment result confirm that the formation of electron channels enhances electron transfer efficiency from bulk to surface, leading to a dual improvement in both electron concentration and reaction activity at surface V_o_ sites, which further promotes the adsorption and activation of O_2_ and NO_2_. Enabled by this strategy, CeO_2_ achieves long‐term stability and new benchmark for ultra‐fast detection of 20 ppb NO_2_ in 5 s at room temperature. This work provides a new strategy to resolve the kinetic contradiction between bulk electron transport and surface reactions.

## Graphical abstract

The formation of electron transport channels overcomes the sluggish electron migration caused by polaron localization, significantly enhancing surface electron concentration and reaction activity, thereby promoting the adsorption and activation of O_2_ and NO_2_. Enabled by this strategy, the CeO_2_‐based sensor achieves ultra‐fast detection of 20 ppb NO_2_ within 5 s at room temperature.

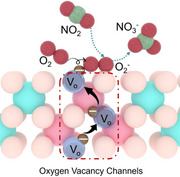

## 1 Introduction

NO_2_ poses a core challenge to industrial safety as a common by‐product and raw material with leakage risks [1]. However, factors like airflow variations and mechanical vibrations in industrial settings can compromise sensor accuracy [2]. Consequently, developing gas sensors capable of rapid response and precise low‐concentration NO_2_ detection is crucial for timely warning and life safety [3, 4, 5]. Although traditional metal oxide semiconductor (MOS) gas sensors offer advantages of low cost and small size, their slow response‐recovery times and baseline drift remain critical drawbacks for practical applications [6, 7, 8]. The reaction mechanism of MOS relies on chemical reactions between electrons at active sites and the target gas [9]. Therefore, enhancing electron transfer efficiency and lifetime to improve surface reaction efficiency and reactivity is essential for developing high‐performance, room‐temperature‐stable NO_2_ sensors.

In recent years, defect engineering has become a key strategy for modulating the band structure and atomic valence states of MOS [10, 11, 12, 13]. Studies have shown that constructing oxygen vacancies (V_o_) in materials such as WO_3_, CeO_2_, CuO, and In_2_O_3_ can generate more localized electrons, enhance surface free electron concentration and molecular adsorption activation capability, thereby improving the gas‐sensing performance of MOS [14, 15, 16, 17]. However, conventional methods are limited to introducing V_o_ only on the surface of MOS, which prevents the timely and continuous replenishment of surface electrons by bulk electrons after participating in reactions [18]. Additionally, isolated V_o_ can act as deep‐level traps, capturing and localizing conduction band electrons, thereby hindering the long‐range delocalized transport of electrons and reducing carrier mobility. Meanwhile, as strongly localized charged defect centers, they exert significant Coulomb scattering effects on migrating charge carriers, increasing scattering probability and thus impairing the electrical conductivity of the MOS. This results in slow sensor response speed and severe baseline drift. Meanwhile, a high concentration of surface V_o_ can reduce the structural stability of reactive sites on the MOS surface, making them prone to sintering or reconstruction under thermodynamic driving forces [19, 20, 21]. This leads to rapid degradation of the gas‐sensing performance of MOS, thereby limiting the service life of MOS‐based sensors. Previous studies have indicated that converting isolated surface V_o_ into surface V_o_ clusters can effectively increase the free electron concentration and enhance the reaction activity of MOS surfaces [22, 23, 24]. This electron‐rich state facilitates the optimization of gas adsorption behavior and reaction pathways and the structure of surface V_o_ clusters is inherently more stable than that of randomly distributed isolated V_o_. However, this strategy remains limited by the fact that V_o_ clusters are primarily confined to the surface region. When substantial electron depletion occurs at the surface, the efficiency of electron replenishment is constrained by the sluggish kinetics of electron migration from the bulk to the surface [25, 26, 27]. This inherent electron transport bottleneck between the surface and the bulk phase hinders the response‐recovery time of the sensor from reaching theoretical limits.

**FIGURE 1:**
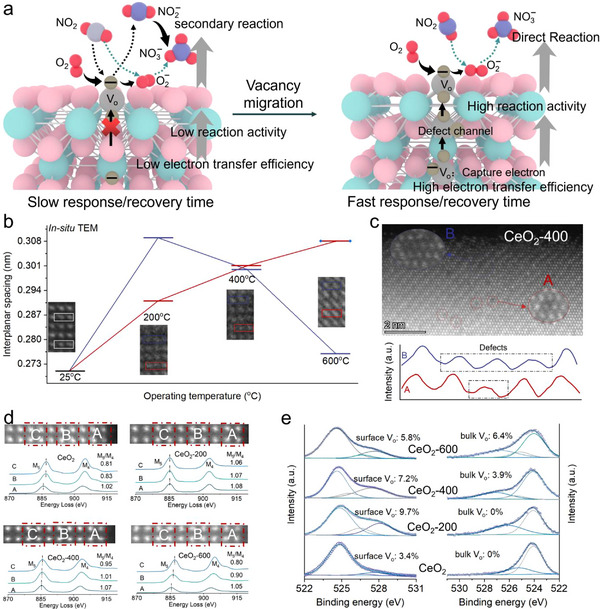
(a) Response mechanism of MOS toward NO_2_ detection. (b) In situ TEM image of CeO_2_. (c) AC‐TEM image of CeO_2_‐400. (d) EELS analysis of CeO_2_, CeO_2_‐200, CeO_2_‐400 and CeO_2_‐600. (e) HAXPES of O2p high‐resolution.

Hence, this study leverages the differential migration energy barriers of surface V_o_ in CeO_2_ to propose a vacuum thermal‐driven method that relocates partially unstable surface V_o_ into the bulk phase and constructs continuous electron transport pathways (Figure 1a). This approach successfully achieves simultaneous enhancement of both response speed and structural stability. The formed electron channels provide an efficient route for electron transfer from bulk V_o_ to the surface V_o_, enabling real‐time replenishment of surface electrons. Experimental results demonstrate that the synergistic improvement in electron lifetime and surface reactivity significantly enhances the long‐term stability and sensitivity of CeO_2_, enabling an ultra‐fast and stable response within 5 s to 20 ppb NO_2_ at room temperature. DFT calculations further confirm that the performance improvement originates from the optimized electron transport mechanism, which enhances the gas adsorption and activation capability of CeO_2_ and significantly reduces the reaction energy barrier. This study elucidates how spatially configured V_o_ govern electron transport, providing enhanced tunability and versatility for gas sensor design.

## 2 Results and Discussion

### 2.1 Structural Characterization of Various Samples

To investigate the evolution of V_o_ under high‐temperature vacuum conditions, we employed comprehensive in situ techniques to examine the changes in crystal structure and surface chemical states of CeO_2_. X‐Ray Diffraction (XRD) patterns and Raman spectra confirm that the crystal structure of CeO_2_ remains intact during the migration process of V_o_ (Figure S1). The influence of V_o_ migration on the crystal structure of CeO_2_ is investigated by in situ transmission electron microscopy (TEM). As shown in Figure 1b, the interplanar spacing of the (200) plane is measured to be 0.271 nm at room temperature. When the temperature reaches 200°C, the interplanar spacings in the edge and center regions become 0.309 and 0.291 nm, respectively. This lattice expansion can be attributed to the formation of positively charged V_o_ from oxygen detachment. To compensate for the charge imbalance, adjacent Ce^4+^ ions are reduced to Ce^3+^, which possess a larger ionic radius. The enhanced repulsion between these ions leads to local lattice expansion and an increase in interplanar spacing. When the temperature is increased to 400°C, the interplanar spacing in the edge region decreases from 0.309 to 0.300 nm, while that in the center region increases from 0.291 to 0.301 nm. This indicates a reorganization of Ce─O bonds in the bulk region, generating additional V_o_, while oxygen atoms migrate toward the surface and fill surface Vo sites. At 600°C, the interplanar spacing in the edge region further decreases, while that in the center region continues to increase.

The presence of vacancies on the CeO_2_‐400 surface is further confirmed by aberration‐corrected transmission electron microscopy (AC‐TEM), which revealed vacancies area in the edge regions is bigger than that of central area (Figure 1c). We further investigated the influence of V_o_ migration on the atomic electronic structure of CeO_2_ using atomic‐resolution monochromatic electron energy‐loss spectroscopy (EELS). As shown in Figure 1d, the analysis is performed across an 8‐atomic layer region at the edge of a CeO_2_ particle. The EELS results of pristine CeO_2_ show that in region‐A, the intensity ratio of the Ce‐M_5_ to Ce‐M_4_ edges is greater than 1, whereas in regions‐B and regions‐C, the ratio is less than 1 and the peak positions shift toward the higher energy‐loss region. This indicates that the valence state of Ce is predominantly Ce^3+^ in region‐A, while it is mainly Ce^4+^ in regions‐B and regions‐C. The free electron concentration in region‐A is also higher than that in regions‐B and regions‐C. In contrast, the escape of oxygen atoms leads to a predominance of Ce^3+^ from region‐A to region‐C in CeO_2_‐200. The increased concentration of surface V_o_ enhances the electron concentration on the CeO_2_ surface. However, the inward migration of V_o_ causes a reduction in surface V_o_ concentration in region‐C of CeO_2_‐400 and in regions‐B and regions‐C of CeO_2_‐600. The EELS results demonstrate that V_o_ in region‐A exhibit high stability and do not migrate under vacuum thermal driving, thereby preserving the electron concentration and reaction activity of the CeO_2_ surface. In contrast, V_o_ in regions‐B and regions‐C are less stable. EDS mapping also confirm that the vacancies migrate inward and eventually form electron channels in the bulk phase under vacuum thermal driving (Figures S2–S5). In situ near‐ambient pressure XPS (NAP‐XPS) is used to track the complex evolution of surface V_o_ concentration in CeO_2_ during vacuum heating. Initially, heating to 200°C caused oxygen release from the surface, leading to a net increase in V_o_ concentration from 3.5% to 6.6%. Subsequently, as the temperature is elevated from 200°C to 400°C, the surface V_o_ concentration declined from 7.2% to 5.2%. A further temperature increases to 600°C eventually caused the concentration to drop to 4.6%. (Figure S6). The spatial distribution of V_o_ in CeO_2_, CeO_2_‐200, CeO_2_‐400 and CeO_2_‐600 are further investigated by hard X‐ray photoelectron spectroscopy (HAXPES). As shown in Figure 1e, the concentration of surface V_o_ and bulk V_o_ in CeO_2_ are 3.4% and 0%, respectively. More notably, the variation in surface V_o_ and bulk V_o_ concentrations across CeO_2_‐200, CeO_2_‐400 and CeO_2_‐600 provides direct evidence that elevating the vacuum annealing temperature drives the inward migration and conversion of surface V_o_ to bulk V_o_. The ration of Ce^3+^/Ce^4+^ in CeO_2_, CeO_2_‐200, CeO_2_‐400 and CeO_2_‐600 also confirm the migration phenomenon of V_o_ (Figure S7). Therefore, by controlling the temperature during vacuum heat treatment, the migration path of V_o_ in CeO_2_ can be regulated, enabling the formation of electron channels and achieving simultaneous optimization of charge carrier transport efficiency and surface reaction activity.

**FIGURE 2:**
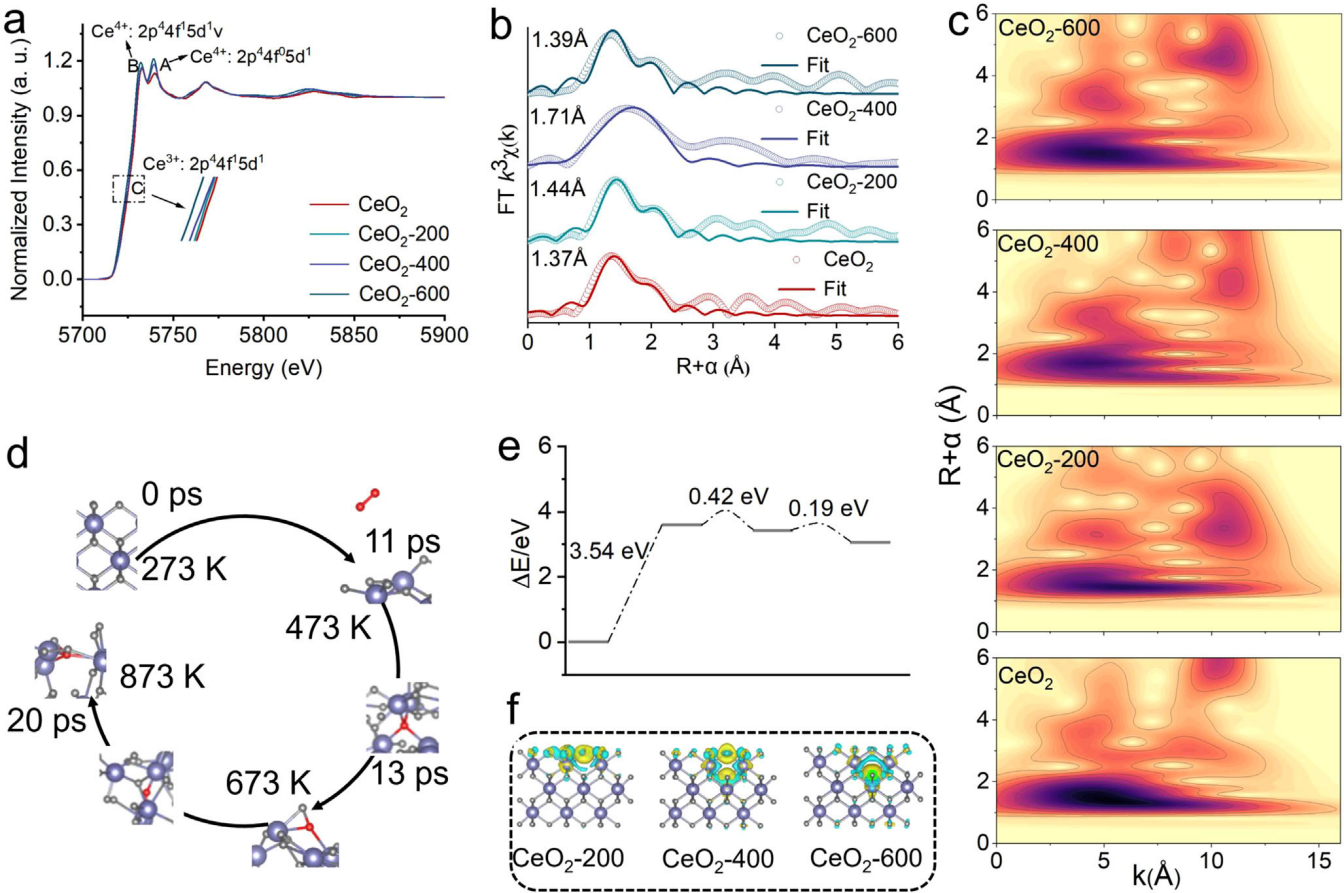
(a) Ce L3‐edge XANES spectra. (b) Ce L3‐edge Fourier‐transformed EXAFS spectra. (c) WT‐EXAFS signals at Ce L3‐edge. (d) MD analysis of CeO_2_. € Reaction barriers of V_o_ migration. f difference charge density of CeO_2_‐200, CeO_2_‐400 and CeO_2_‐600.

X‐ray absorption near‐edge structure (XANES) measurements are performed to probe the valence evolution of Ce in response to vacuum thermal treatment. The characteristic spectral features include peaks A and B, which are attributed to Ce^4+^ and correspond to final states of 2p^4^4f^0^5d^1^ and 2p^4^4f^1^5d^1^v respectively, while peak C is assigned to Ce^3+^ with a final state of 2p^4^4f^1^5d^1^. A distinct redshift is observed in CeO_2_‐200 compared to CeO_2_, indicating an increased Ce^3+^ concentration due to surface V_o_ detachment. In contrast, CeO_2_‐400 exhibits a blueshift relative to CeO_2_‐200, suggesting that the migration of sub‐surface oxygen atoms to the surface results in the annihilation of surface V_o_. Furthermore, CeO_2_‐600 shows a redshift compared to CeO_2_‐400, which is attributed to the predominant formation of bulk V_o_ at the elevated temperature of 600°C (Figures 2a; S8). The fitting results of the extended X‐ray absorption fine structure (EXAFS) and the corresponding wavelet‐transform patterns (Figure 2b,c) further corroborate this dynamic evolution. Compared with the CeO_2_‐200, the apparent discrepancy between the XANES blue shift and the elongated Ce─O bond length in EXAFS for CeO_2_‐400 is attributed to the temperature‐induced redistribution of oxygen vacancies from the surface to the bulk. This process increases the average Ce oxidation state while simultaneously introducing more pronounced lattice strain in the bulk, leading to longer average bond lengths. The data confirm that V_o_ are initially generated on the surface, then migrate through the sub‐surface region and ultimately propagate into the bulk phase as the treatment temperature increases from 200°C to 600°C (Figure S9).

To investigate the formation mechanism of electron channels, we employed a combined approach using molecular dynamics (MD) and density functional theory (DFT). As shown in Figure 2d, oxygen atoms exhibit a clear transport behavior with increasing temperature. Initially, as the temperature rises from 0 K to 473 K, a surface oxygen atom escapes from CeO_2_ surface. Upon further heating to 673 K, Ce─O bonds break in the bulk region and the released oxygen atom migrates to the surface, bonding with a surface Ce atom. Ultimately, an additional oxygen atom from the bulk migrates to the surface and again bonds with a surface Ce atom, completing a second migration cycle at 873 K. The energy changes during V_o_ migration are further examined via DFT. The reaction barrier for the breaking of surface Ce─O bonds and the escape of an oxygen atom is 3.54 eV. The subsequent cleavage of Ce─O bonds in the bulk region has a reaction barrier of 0.42 eV. The oxygen atom then migrates to the surface and spontaneously forms a Ce─O bond. The rupture of another bulk Ce─O bond has a barrier of 0.19 eV and the corresponding oxygen atom also spontaneously forms a new Ce─O bond on the surface (Figure 2e). Both MD and DFT results indicate that as temperature increases, internal Ce─O bonds begin to break, while the migration of bulk oxygen atoms to the surface and the subsequent formation of Ce─O bonds occur spontaneously without requiring external energy input. The charge density difference of CeO_2_‐200, CeO_2_‐400 and CeO_2_‐600 are shown in Figure 2f. In CeO_2_‐200 and CeO_2_‐600, the electron accumulation regions are predominantly confined to either the surface or the bulk, respectively. In contrast, CeO_2_‐400 exhibits a distinct electronic structure where computed charge distribution suggests the formation of interconnected electron accumulation pathways bridging the surface and bulk regions. This configuration facilitates continuous charge exchange between the bulk and the surface. This unique feature in CeO_2_‐400 enables efficient electron replenishment to the surface active sites, potentially enhancing its reaction activity compared to the other two samples.

### 2.2 Gas Performance of Detecting NO_2_ at Room Temperature

**FIGURE 3:**
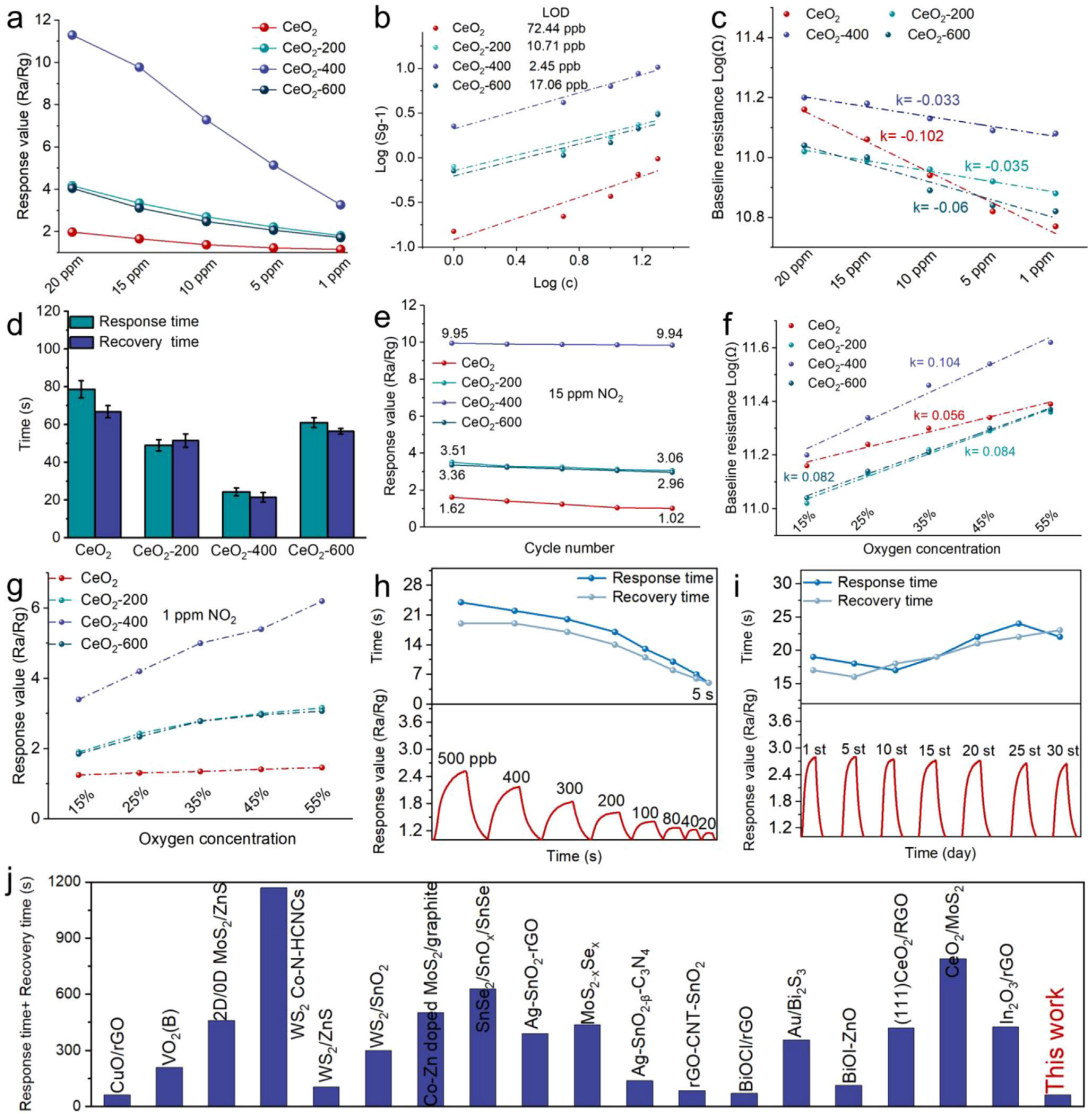
(a) Response values. (b) Response‐concentration correlation. (c) Baseline resistance. (d) Response‐recovery time. (e) 5‐cycle stability response. (f,g) Baseline resistance and response value at different concentration oxygen. (h) Response values of CeO_2_‐400 toward low concentration NO_2_. (i) Long‐term stability test. j response‐recovery time of this work compared with previous works. (Date adapted with permission from Springer Nature, John Wiley and Sons, American Chemistry Society and Elsevier).

**FIGURE 4:**
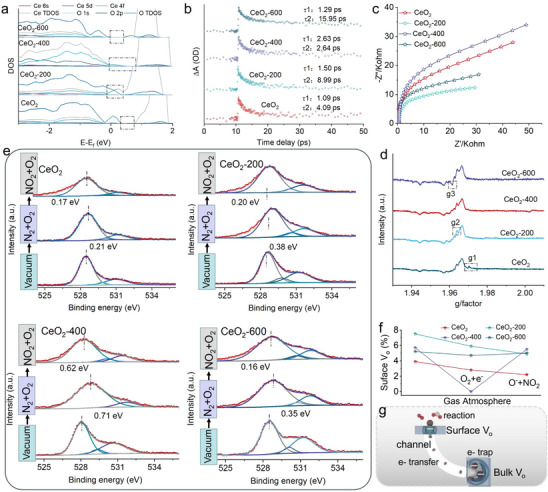
(a) DOS analysis of CeO_2_, CeO_2_‐200, CeO_2_‐400 and CeO_2_‐600. (b) Electron decay lifetime. (c) EIS analysis. (d) EPR analysis. (e) In situ NAP‐XPS analysis of CeO_2_, CeO_2_‐200, CeO_2_‐400 and CeO_2_‐600 under O_2_ and NO_2_. (f) Proportional distribution of V_o_ valence states in each treatment from the peak‐differentiating analysis of NAP‐XPS results. g Electron transfer mechanism.

The enhancement of electrons transfer efficiency from bulk to surface improve the reaction activity of CeO_2_‐400. To further assess its practical applicability, we systematically evaluated its response‐recovery time, dynamic response characteristics, and long‐term stability. The sensing layers of CeO_2_‐400 is authenticated to be 115 µm thick (Figure S10). The response values of CeO_2_, CeO_2_‐200, CeO_2_‐400 and CeO_2_‐600 to various concentrations of NO_2_ are shown in Figure 3a. The response of CeO_2_‐200 to NO_2_ is higher than that of pristine CeO_2_, which can be attributed to the increased concentration of V_o_. In comparison, CeO_2_‐600 exhibits a slightly lower response than CeO_2_‐200, likely due to its reduced specific surface area (Figure S11). When the concentrations of surface and bulk V_o_ reach equilibrium, the bulk V_o_ provide efficient pathways for oxygen migration and charge transport, achieving optimal kinetic matching with the gas adsorption and reaction sites dominated by surface V_o_. Furthermore, all samples exhibit a positive linear response to NO_2_ within the tested concentration range. Using the Root Mean Square Deviation (RMSD) method, the theoretical limits of detection (LOD) for CeO_2_, CeO_2_‐200, CeO_2_‐400 and CeO_2_‐600 are determined to be 72.44 ppb, 10.71 ppb, 2.45 ppb and 17.06 ppb, respectively. As a result, CeO_2_‐400 demonstrates both a higher response value and a superior theoretical detection limit compared to CeO_2_, CeO_2_‐200 and CeO_2_‐600 (Figure 3b). The baseline resistance of the sensing layers for all samples is shown in Figure 3c. The baseline resistance of CeO_2_ increases significantly with rising NO_2_ concentration, thereby reducing its detection accuracy. The introduction of surface V_o_ in CeO_2_‐200 enhances surface reactivity and suppresses the baseline resistance drift. When some surface V_o_ are transformed into bulk V_o_, the formation of electron transfer channels improves electron transport efficiency from bulk to surface, further inhibiting baseline resistance drift in CeO_2_‐400. Conversely, excessive conversion of surface V_o_ to bulk V_o_ in CeO_2_‐600 reduces the surface free electron concentration and reactivity, leading to increased baseline drift. Compared with previously reported semiconductor‐based NO_2_ gas sensors, CeO_2_‐400 exhibits a faster response‐recovery time (Figure 4j). Additionally, the baseline resistance of CeO_2_‐400 remains stable at room temperature. Therefore, CeO_2_‐400 shows great potential for practical applications. The effect of electron transfer channels on response‐recovery times is illustrated in Figure 3d; Figure S12. The response and recovery times of CeO_2_ are 78 s and 66 s, respectively, while the introduction of surface V_o_ shortens these values to 48 s and 52 s. The balance between electron replenishment and surface electron consumption in CeO_2_‐400 modifies the surface properties and enhances adsorption activation, further reducing the response time. The cycling stability of the samples is evaluated over five response‐recovery cycles toward 15 ppm NO_2_ at room temperature (Figures 3e; S13). The low surface activity of CeO_2_ leads to the accumulation of intermediate products during reactions, resulting in a gradual decrease in response over multiple cycles. The introduction of surface V_o_ improves the cycling stability of CeO_2_‐200. The formation of electron transfer channels further enhances electron transfer efficiency from bulk to surface, making the cycling stability of CeO_2_‐400 superior to that of CeO_2_‐200. However, excessive conversion of surface V_o_ to bulk V_o_ in CeO_2_‐600 reduces surface adsorption capacity and reaction activity, leading to deteriorated cycling stability.

The reaction mechanism between active species and oxygen molecules is further investigated by evaluating the gas‐sensing performance under different oxygen concentrations. The baseline resistance of all samples increased with rising oxygen concentration and the rate of resistance change for CeO_2_‐400 is greater than that of CeO_2_, CeO_2_‐200 and CeO_2_‐600. This indicates that CeO_2_‐400 can generate more free electrons to react with oxygen molecules, thereby producing more active oxygen species (Figure 3f). Consequently, the variation in response values of CeO_2_‐400 under different oxygen concentrations is also more pronounced compared to CeO_2_, CeO_2_‐200 and CeO_2_‐600 (Figure 3g). The dynamic response curve of CeO_2_‐400 toward low‐concentration NO_2_ is shown in Figure 3h. The response value of CeO_2_‐400 to 20 ppb NO_2_ is 1.18, indicating that the sensor is capable of detecting concentrations below the EPA limit (53 ppb). Furthermore, long‐term stability of CeO_2_‐400 toward NO_2_ detection is shown in Figures 3i and S14. Both the response value and response time of CeO_2_‐400 exhibited only minor deviations compared to the initial measurements, demonstrating excellent long‐term stability. In addition, the baseline resistance of CeO_2_‐400 remained stable at room temperature. In general, humidity can influence the gas‐sensing performance of MOS as the operating temperature is room temperature. The mechanistic understanding of NO_2_ detection by CeO_2_‐400 under humid conditions was further explored. As shown in Figure S15, the response of all samples toward NO_2_ decreased with increasing relative humidity. This decline can be attributed to a competitive adsorption mechanism. Although the abundant electron channels formed on the surface of CeO_2_‐400 enhance gas sensing performance, they also facilitate the adsorption of water molecules at active sites. Water vapor and NO_2_ molecules therefore compete for the same adsorption sites. As humidity rises, the growing coverage of surface‐adsorbed water and hydroxyl groups progressively blocks these active sites, inhibiting the adsorption and subsequent reaction of NO_2_ and resulting in the observed reduction in sensor response. The selectivity performance of CeO_2_‐400 is shown in Figure S16. At 10 ppm concentration of various target gas molecules, the response value of various sample for the detection of NO_2_ is better than those for the detection of other gas molecules. Compared with previously reported semiconductor‐based NO_2_ gas sensors, CeO_2_‐400 exhibits a faster response‐recovery time (Figure 3j). Additionally, the baseline resistance of CeO_2_‐400 remains stable at room temperature, which shows great potential for practical applications.

### 2.3 Surface Reaction Mechanism and Density Functional Theory Calculations

The density of states (DOS) analysis confirms that the introduction of surface V_o_ in CeO_2_‐200 gives rise to defect levels within the band gap and promotes the formation of a hybridized state between Ce‐4f and O‐2p orbitals. This hybridization reduces the electron transfer energy and enhances the capacity for generating active oxygen. As some surface V_o_ transform into bulk V_o_, the defect states associated with these two spatially separated V_o_ are situated at distinct positions near the Fermi level. As a result, the overlap and hybridization between their wavefunctions are minimal. Consequently, no hybridized state is detected within the bandgap of CeO_2_‐400 and CeO_2_‐600. Nevertheless, the existence of bulk V_o_ still narrows the band gap, thereby facilitating electron transfer (Figure 4a). The electrons transfer mechanism at room temperature is explored via MIR‐TAS. The relaxation time of free carriers varies are different due to the presence of trapped states. The multiexponential fitting of the excited‐state absorption signals reveals two distinct decay lifetimes for CeO_2_, CeO_2_‐200, CeO_2_‐400 and CeO_2_‐600, corresponding to electron trapping at surface defects and recombination processes in the bulk. The lifetime components of CeO_2_ are 1.09 ps (τ1) and 4.09 ps (τ2), with amplitude weights of A1 and A2 are 0.993 and 0.007. These results indicate that electron decay in CeO_2_ is dominated by the trapping of electrons at surface V_o_, with no significant contribution from bulk recombination, confirming that V_o_ are primarily located on the surface. Similarly, CeO_2_‐200 exhibits lifetimes of 1.50 ps (τ1) and 8.99 ps (τ2), with amplitude weights of A1 and A2 are 0.993 and 0.007, suggesting that electron decay also proceeds mainly via trapping at surface V_o_. In contrast, CeO_2_‐400 shows lifetimes of 2.63 ps (τ1) and 2.64 ps (τ2), with amplitude weights of A1 and A2 are 0.643 and 0.357. This indicates that electron decay occurs through two competing pathways, namely trapping by surface V_o_ and recombination via bulk V_o_. Meanwhile, CeO_2_‐600 presents lifetimes of 1.29 ps (τ1) and 15.95 ps (τ2), with amplitude weights of A1 and A2 are 0.514 and 0.486, confirming a stronger contribution from bulk V_o_ to electron decay compared to CeO_2_‐400. Additionally, CeO_2_‐400 possesses a longer average electron lifetime than CeO_2_, CeO_2_‐200 and CeO_2_‐600 (Figure 4b). The electrochemical impedance spectroscopy (EIS) results show that the diameter of the Nyquist plot for CeO_2_‐400 is bigger than those of CeO_2_, CeO_2_‐200 and CeO_2_‐600, indicating that CeO_2_‐400 possesses the highest barrier and the most active chemical state. When NO_2_ reacts with the active oxygen species, it can more effectively inject electrons into CeO_2_‐400, significantly reducing the surface barrier and thereby generating extremely high sensitivity. (Figure 4c).

Electron paramagnetic resonance (EPR) analysis further confirms that heat treatment promotes the conversion of high‐coordinated, low‐activity Ce^3+^‐g1 on the CeO_2_ surface into low‐coordinated, highly active Ce^3+^‐g2, which enhances the reaction efficiency between free electrons and O_2_ molecules (Figure 4d). The reaction process between NO_2_ and O_2_ in all samples is investigated by in situ NAP‐XPS (Figure 4e). At room temperature, the reaction of electrons with O_2_ in CeO_2_‐400 to produce active oxygen causes the peaks of surface V_o_ to shift from 528.08 to 528.79 eV and the concentration of surface V_o_ decreases from 5.7% to 0%. Subsequently, when NO_2_ is introduced into the reaction chamber, the peaks of surface V_o_ in CeO_2_‐400 shift from 528.79 eV to 528.17 eV and the V_o_ concentration increases from 0% to 5.5%. Similarly, under room temperature conditions, the introduction of O_2_ and NO_2_ also induces shifts in the surface V_o_ peaks in CeO_2_, CeO_2_‐200 and CeO_2_‐600. However, in CeO_2_‐400, the surface V_o_ peak disappears during the conversion of O_2_ molecules into active oxygen. This suggests that the formation of an electron transfer channel enhances the efficiency of electron transfer from bulk V_o_ to surface V_o_ sites. Additionally, the differences in binding energy shifts of CeO_2_, CeO_2_‐200, CeO_2_‐400 and CeO_2_‐600 during the reaction process demonstrate that the formation of electron channels enhances the surface free electron concentration. Therefore, the improved surface reaction activity subsequently lowers the energy barrier for the conversion of O_2_ to active oxygen, leading to the complete occupancy of surface V_o_ sites by active oxygen. When NO_2_ enters the reaction chamber, the surface V_o_ peak in CeO_2_‐400 reappears and shifts to lower binding energy. In contrast, the conversion of O_2_ to active oxygen does not cause the disappearance of surface V_o_ in CeO_2_, CeO_2_‐200 and CeO_2_‐600. Furthermore, during the reaction between active oxygen and NO_2_, the surface V_o_ concentration in CeO_2_, CeO_2_‐200 and CeO_2_‐600 does not return to near its initial level (Figure S17). This indicates that the formation of electron migration channels in CeO_2_‐400 not only accelerates electron transfer efficiency from bulk to surface but also supplies sufficient lattice oxygen to the surface to participate in the reaction, thereby improving the long‐term stability of surface V_o_ and ultimately enabling rapid and stable detection of NO_2_ (Figure 4f). The electron transfer mechanism is schematically illustrated in Figure 4g. The evolution from isolated surface V_o_ to a bulk‐connected 3D percolating network drives a dual and synergistic physical transformation. On one hand, the electronic ground state shifts from localized small polarons to delocalized network‐extended states, fundamentally altering the conduction mechanism. On the other hand, randomly distributed strong scattering potential wells are reconfigured into a preferential conduction channel with a smoothed potential barrier, which significantly reduces the effective electron scattering cross‐section through a collective shielding effect. This spatial reconstruction essentially transforms V_o_ from scattering sources into efficient conduction pathways. Consequently, it simultaneously enhances carrier concentration and substantially increases carrier mobility, thereby synergistically optimizing the surface electron concentration of CeO_2_. It is noteworthy that the highest interfacial resistance of CeO_2_‐400 in air, reflects its pronounced surface barrier—a prerequisite for achieving high sensitivity by providing a wide electrical signal modulation range. The engineered V_o_ channels do not aim to lower this steady‐state resistance but are dedicated to addressing the kinetic bottleneck of electron consumption during surface reactions. They serve as efficient pathways for the rapid and directional replenishment of electrons from the bulk reservoir to the surface active sites upon gas exposure, ensuring that the high barrier can be modulated both substantially and swiftly.

**FIGURE 5:**
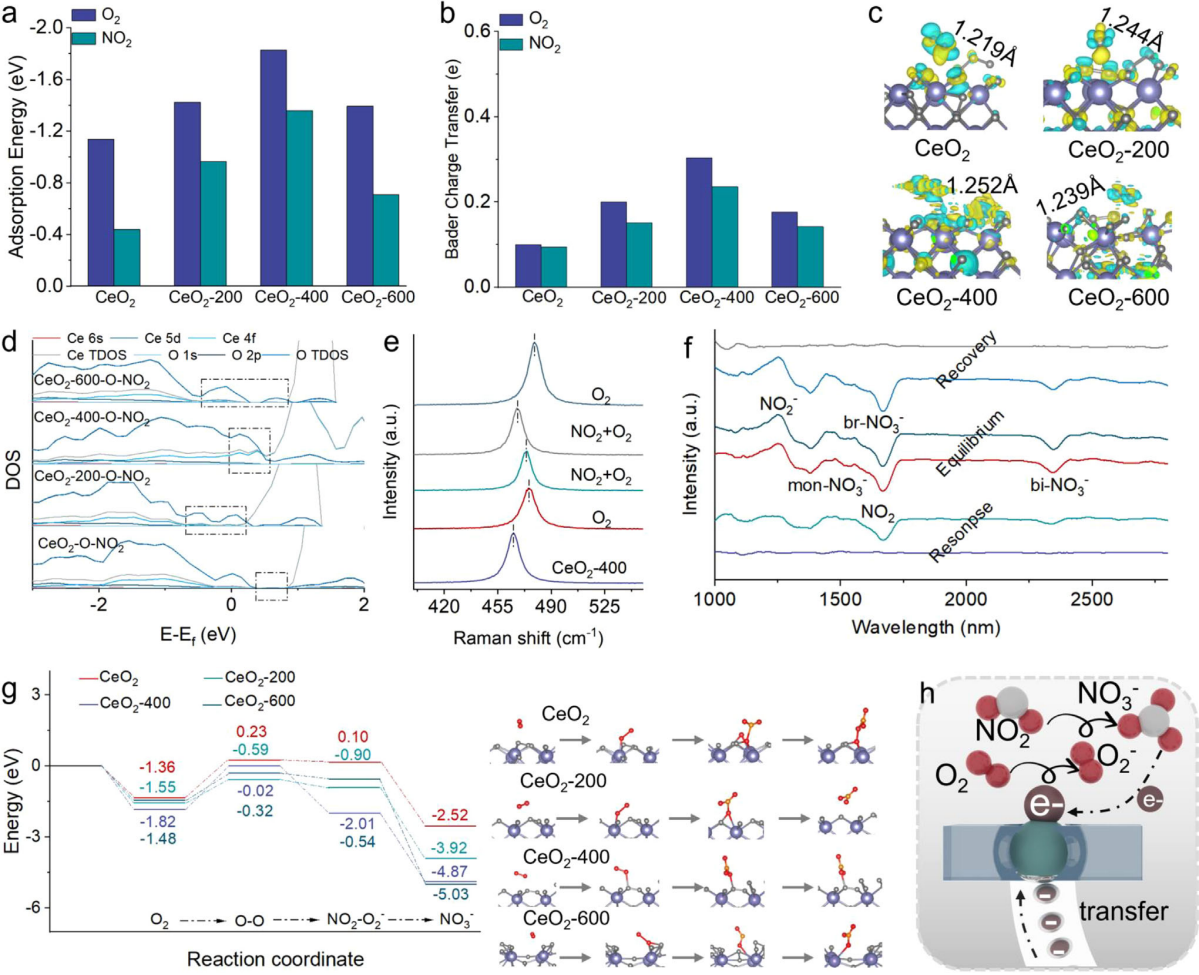
(a) Adsorption energy of CeO_2_, CeO_2_‐200, CeO_2_‐400 and CeO_2_‐600 toward O_2_ and NO_2_. (b) Bader analysis of charge transfer amount. (c) Charge difference distributions of CeO_2_, CeO_2_‐200, CeO_2_‐400 and CeO_2_‐600 toward O_2_. (d) DOS analysis toward activity oxygen and NO_2_ reaction. (e) In situ Raman spectra. (f) In situ FTIR spectra of CeO_2_‐400. (g) Reaction energy (Ea) and reaction model for the reaction of NO_2_ with activity oxygen. (h) Diagram of reaction process.

To understand the influence of electron transfer channel on the adsorption and activation of molecules on the CeO_2_ surface, DFT calculations are employed to analyze the adsorption energy of various samples in response to O_2_ and NO_2_ (Figures 5a; S18). The adsorption energies of CeO_2_, CeO_2_‐200, CeO_2_‐400 and CeO_2_‐600 toward O_2_ are −1.14, −1.42, −1.82 and −1.39 eV, respectively. When active oxygen is already present on the surface, the adsorption energies of CeO_2_, CeO_2_‐200, CeO_2_‐400 and CeO_2_‐600 toward NO_2_ are −0.44, −0.96, −1.35 and −0.71 eV, respectively. The differential charge density and bader electron analysis of all samples toward O_2_ and NO_2_ are shown in Figure 5b. When oxygen molecules adsorb on the surface of all samples, significant electron accumulation and dissipation occur. The bader charge transfer amounts between O_2_ and CeO_2_, CeO_2_‐200, CeO_2_‐400 and CeO_2_‐600 are 0.10 e, 0.20 e, 0.31 e and 0.18 e. The bader charge transfer amounts between NO_2_ and CeO_2_, CeO_2_‐200, CeO_2_‐400 and CeO_2_‐600 are 0.09 e, 0.15 e, 0.24 e and 0.14 e, respectively. The charge difference distributions of CeO_2_, CeO_2_‐200, CeO_2_‐400 and CeO_2_‐600 toward O_2_ is shown in Figure 5c. During the adsorption‐activation of O_2_ process, significant charge accumulation occurs inside CeO_2_‐400 and the electron exchange between its surface V_o_ sites and O_2_ is markedly greater than that of CeO_2_, CeO_2_‐200 and CeO_2_‐600. This indicates that the bulk V_o_ in CeO_2_‐400 can capture electrons and transfer them to the surface V_o_, effectively replenishing the electron concentration at the surface. As a result, both strong electron accumulation and dissipation coexist at the surface V_o_ sites. Furthermore, the O‐O bond length confirms that CeO_2_‐400 exhibits significantly superior O_2_ adsorption and activation capabilities compared to CeO_2_, CeO_2_‐200, and CeO_2_‐600. The adsorption energy and Bader charge result confirm that the formation of electrons transfer channel improve electron transfer efficiency from bulk to surface during the generation of active oxygen, which enhance the surface reaction activity. The projected density of states (PDOS) for activity oxygen and NO_2_ reaction process result further confirms that the formation of bulk V_o_ and surface V_o_ promotes the generation of multiple defect level, which reduce the energy required to excite electrons from the O 2p state to the Ce 4f state (Figure 5d). Additionally, the establishment of electron transfer channels promotes the electron accumulation at surface V_o_ sites, thereby inducing significant electron exchange with CeO_2_ during the NO_2_ and activity oxygen reaction. Consequently, the detection efficiency of CeO_2_‐400 toward NO_2_ is significantly superior to that of CeO_2_, CeO_2_‐200 and CeO_2_‐600 (Figure S14). Then, the surface reaction mechanism in CeO_2_‐400 is deeply explored via in situ Raman (Figure 5e). The peak at 464.7 cm^−1^ is typical F2g of CeO_2_‐400. The blue shift of the F2g peak of CeO_2_‐400 occurs in O_2_ atmosphere due to lattice shrinkage and oxygen vacancies consumption, resulting in two Ce^4+^ ions (0.97 Å) replacing two Ce^3+^ ions (ionic radius 1.14 Å) [28]. Then, the introduction of NO_2_ in reaction chamber caused the phenomenon of red shift in F2g peak, which confirmed that the reaction of NO_2_ with O_2_ can produce electrons leading to the reduction of Ce^4+^ to Ce^3+^. In situ Raman spectra result also confirm that the adsorption‐reaction sites on the CeO_2_‐400 surface are surface V_o_ and the shift of the F2g peak demonstrates the excellent stability of the surface V_o_ sites in CeO_2_‐400.

In situ FTIR spectroscopy is employed to investigate the reaction of NO_2_ on the surface of all samples at room temperature. As shown in Figure 5f, the surface reaction of CeO_2_‐400 during NO_2_ exposure can be divided into three stages. During the response stage, the peak at 1664 cm^−1^ is attributed to adsorbed NO_2_ and the absorbance intensity continuously increases. Simultaneously, the peaks emerge at 1260 cm^−1^, 1452 cm^−1^ and 2345 cm^−1^, corresponding to the formation of NO_2_^−^, mon‐NO_3_^−^ and bi‐NO_3_^−^, respectively [29]. During the equilibrium stage, the peaks assigned to NO_2_^−^ and mon‐NO_3_^−^ further increase in intensity, while the intensity of the bi‐NO_3_^−^ peak decreases. This indicates that the reaction between NO_2_ and active oxygen proceeds steadily toward a steady state, leading to significant product accumulation. Additionally, a peak at 1550 cm^−1^ appears, which is ascribed to br‐NO_3_^−^ [30]. During the recovery stage, the absorbance signals of NO_2_^−^, mon‐NO_3_^−^, br‐NO_3_^−^ and bi‐NO_3_^−^ continuously decrease until they disappear, confirming the complete desorption of these species from the CeO_2_ surface. This behavior reflects the excellent recyclability of CeO_2_‐400, consistent with its stable baseline resistance and reliable gas‐sensing performance (Figure S15). Therefore, based on the in situ FTIR results, the detection process of NO_2_ can be described in three steps as shown in the following equations. First, NO_2_ molecules undergo physical adsorption on the CeO_2_ surface. Then, the adsorbed NO_2_ reacts with O_2_^−^ to form NO_3_^−^ and release a gaseous oxygen atom. During the reaction, as NO_2_ is converted into NO_3_^−^, the adsorbed O_2_^−^ species is consumed, leading to the partial release of electrons back into the conduction band, thereby reducing the resistance of the n‐type semiconductor [31, 32]. Finally, under ambient humidity, the surface of the sensing material typically possesses abundant H_2_O. The NO_3_^−^ can react with adjacent surface H_2_O to form gaseous HNO_3_ and OH^−^ [33, 34].

$$O_{2}+e^{-}\to {O_{2}}^{-}$$

$$NO_{2}\left(gas\right)\to NO_{2}\left(ads\right)$$

$$2NO_{2}\left(ads\right)+{O_{2}}^{-}\to 2N{O_{3}}^{-}\left(ads\right)+e^{-}$$

$$N{O_{3}}^{-}\left(ads\right)+H_{2}O\to HNO_{3}+OH^{-}$$

The energy barriers of the reaction processes are further studied using DFT calculations (Figure 5g). Initially, adsorbed O_2_ molecules react with electrons to spontaneously generate O_2_^−^ on the surfaces of the CeO_2_ materials. The energy barriers for O_2_ adsorption on CeO_2_, CeO_2_‐200, CeO_2_‐400 and CeO_2_‐600 are −1.35 eV, −1.55 eV, −1.82 eV and −1.48 eV, respectively. The lower energy barriers of CeO_2_‐200, CeO_2_‐400 and CeO_2_‐600 compared to CeO_2_ suggest that the formation of electron transfer channels promotes the generation of more active oxygen species. Subsequently, the active oxygen species react spontaneously with NO_2_ to form NO_3_^−^. The corresponding reaction barriers for CeO_2_, CeO_2_‐200, CeO_2_‐400, and CeO_2_‐600 are 0.10 eV, −0.90 eV, −2.01 eV and 0.54 eV, indicating that asymmetric V_o_ also enhance the interaction between NO_2_ and CeO_2_‐400. Thus, the significantly improved NO_2_ detection performance can be ascribed to the reduce of reaction barriers. Accordingly, the surface reaction mechanism of CeO_2_‐400 toward NO_2_ is proposed in Figure 5h. The formation of electron transfer channels increases the electron concentration at surface V_o_ sites, thereby promoting the generation of active oxygen species and enabling deep oxidation of NO_2_ to NO_3_^−^. As a result, CeO_2_‐400 exhibits both rapid response and long‐term stability in gas‐sensing applications.

## 3 Conclusion

In summary, this work leverages the difference in migration barriers of surface V_o_ in CeO_2_ to achieve directional migration of surface V_o_ into the bulk via precisely controlled vacuum thermal treatment, thereby constructing interconnected electron transport channels bridging the bulk and surface regions. The formation of electron channel effectively mitigates the issues of slow response‐recovery time and baseline resistance drift phenomenon during NO_2_ detection. Through a combination of in situ characterization and theoretical calculations, we confirmed that the electron channels significantly enhance both electron concentration and reactivity at surface V_o_ sites, strengthening the adsorption‐activation ability toward O_2_ and NO_2_, while considerably reducing the reaction energy barrier between NO_2_ and active oxygen species. The optimized CeO_2_‐400 exhibits outstanding NO_2_ sensing performance at room temperature, demonstrating an ultra‐fast response time of 5 s toward 20 ppb NO_2_ and excellent long‐term stability. Furthermore, the established electron transfer channels facilitate the generation of surface active oxygen species, promoting deep oxidation of NO_2_ to NO_3_^−^ and effectively suppressing secondary reaction. This work not only resolves the long‐standing kinetic mismatch between bulk electron transport and surface reactions in metal oxide gas sensors, but also establishes a universal paradigm for designing high‐performance functional materials via defect‐structure engineering for applications in gas sensor, catalysis and energy conversion.

## Conflicts of Interest

The authors declare no conflict of interest.

## Supporting information




**Supporting File**: advs73831‐sup‐0001‐SuppMat.doc.

## Data Availability

The data that support the findings of this study are available from the corresponding author upon reasonable request.

## References

[1] P. Garrido‐Barros , J. Derosa , M. J. Chalkley , and J. C. Peters , “Tandem electrocatalytic N2 fixation via proton‐coupled electron transfer,” Nature 609 (2022): 71–76. 36045240 10.1038/s41586-022-05011-6 PMC10281199

[2] H. Lim , H. Kwon , H. Kang , J. E. Jang , and H. J. Kwon , “Semiconducting MOFs on ultraviolet laser‐induced graphene with a hierarchical pore architecture for NO2 monitoring,” Nature Communication 14 (2023): 3114. 10.1038/s41467-023-38918-3 PMC10229625 37253737

[3] W. J. Quan , J. Shi , H. Y. Luo , et al., “Fully Flexible MXene‐based Gas Sensor on Paper for Highly Sensitive Room‐Temperature Nitrogen Dioxide Detection,” ACS Sensors 2023, 8, 103–113. 36635889 10.1021/acssensors.2c01748

[4] Z. X. Wu , H. Wang , Q. L. Ding , et al., “A Fiber Sensor for VOC Gas Detection Based on Modulated Aggregation of Fluorescence Indicator,” Advance Functional Material 33 (2023): 202300046.

[5] Z. Dong , Q. Hu , H. Liu , et al., “3D flower‐like Ni doped CeO2 based gas sensor for H2S detection and its sensitive mechanism,” Sensors Actuators B‐Chemical 357 (2022): 131227.

[6] S. Zhou , L. Yao , H. Mei , M. Lu , L. Cheng , and L. Zhang , “Strengthening PPy/TiO2 arrayed SiOC honeycombs for self‐protective gas sensing,” Composites Part B: Engineering 230 (2022): 109536.

[7] S. Zhou , L. Yao , T. Zhao , et al., “Chemiresistively sensitized SiOC structure for formaldehyde detection under thermal and pressure loading,” Carbon 201 (2023): 100–109.

[8] X. L. Cheng , Y. Z. Liu , W. Zhong , et al. Advance Science 12 (2025): 202509293.

[9] Y. C. Ou , B. Wang , N. A. Xu , et al. Advance Science 11 (2024): 202402038.

[10] H. C. Zhao , J. Li , X. P. She , et al. ACS Sensors 9 (2024): 2183–2193. 38588327 10.1021/acssensors.4c00382

[11] Y. J. Zhang , Y. D. Jiang , Z. Yuan , et al. Small 19 (2023): 202303631.

[12] K. S. Pasupuleti , A. M. Thomas , D. Vidyasagar , et al., “ZnO@Ti 3 C 2 T x MXene Hybrid Composite‐Based Schottky‐Barrier‐Coated SAW Sensor for Effective Detection of Sub‐ppb‐Level NH 3 at Room Temperature under UV Illumination,” ACS Materials Letters 5 (2023): 2739–2746.

[13] L. Zhao , C. C. Yu , C. C. Xin , et al. Advance Functional Materials 34 (2024): 202314174.

[14] N. Luo , C. Wang , D. Zhang , et al., “Ultralow detection limit MEMS hydrogen sensor based on SnO2 with oxygen vacancies,” Sensors Actuators B‐Chemical 354 (2022): 130982.

[15] Q. Hu , H. Jiang , W. Zhang , X. Wang , X. Wang , and Z. Zhang , “Unveiling the synergistic effects of hydrogen annealing on CeO2 nanofibers for highly sensitive acetone gas detection: Role of Ce3+ ions and oxygen vacancies,” Applied Surface Science 640 (2023): 158411.

[16] X. Liu , D. Yang , Y. Guo , et al., “Room temperature triethylamine gas sensor with excellent performances based on novel porous CuO foam via a simple and facile route,” Sensors Actuators B‐Chemical 390 (2023): 133934.

[17] Y. Y. Huo , L. M. Qiu , T. Q. Wang , et al., ACS Sensors 9 (2024): 3433–3443. 38872232 10.1021/acssensors.4c00866

[18] X. Wang , Y. Li , X. Jin , G. Sun , J. Cao , and Y. Wang , “The effects of Co doping on the gas sensing performance of In2O3 porous nanospheres,” Sensors Actuators B‐Chemical 403 (2024): 135155.

[19] J. X. Li , X. C. Mo , C. H. Zhu , and M. H. Yang Advanced Sensor Research 4 (2025): 202400192.

[20] Z. C. Zheng , K. W. Liu , Y. W. Zhou , et al. Advanced Science 11 (2024): 202408096.

[21] S. Xiao , Y. H. Xue , Z. Y. Li , et al. ACS Sensors 10 (2025): 2976–2985. 40131740 10.1021/acssensors.4c03741

[22] M. Wei , P. Zhang , S. Zhou , X. Wang , G. Wang , and J. Zhao , “Vacancy clustering behaviors and stable configurations in vanadium metal: First‐principles investigations,” Nuclear Material and Energy 33 (2022): 101296.

[23] Y. Y. Zhang , X. S. Kong , G. Z. Feng , L. Chen , C. Zhang , and G. Zhao , “Energetic and structural stability of vacancy clusters in Al under external stress conditions,” Computational Material Science 248 (2025): 113562.

[24] E. C. Shin , Y. Kang , and S. H. Jeon , “Nitrogen Complex‐Driven Vacancy Cluster in Group‐III Nitrides,” ACS Applied Material and Interfaces 16 (2024): 64244–64252. 10.1021/acsami.4c13648 39499806

[25] Z.‐Y. Wu , P. Zhu , D. A. Cullen , et al., “A general synthesis of single atom catalysts with controllable atomic and mesoporous structures,” Nature Synthesis (2022): 658–667.

[26] F. Lu , D. Yi , S. Liu , et al., “Engineering Platinum–Oxygen Dual Catalytic Sites via Charge Transfer towards Highly Efficient Hydrogen Evolution,” Angewandte Chemie International Edition 59 (2020): 17865–17871. 10.1002/anie.202008117 32621558

[27] Y. Zhang , J. Zhao , H. Wang , et al., “Author Correction: Single‐atom Cu anchored catalysts for photocatalytic renewable H2 production with a quantum efficiency of 56%,” Nature Communication 13 (2022): 2062. 10.1038/s41467-022-29799-z PMC9001637 35411001

[28] J. A. Sullivan , P. Dulgheru , I. Atribak , A. Bueno‐López , and A. García‐García , “Attempts at an in situ Raman study of ceria/zirconia catalysts in PM combustion,” Applied Catalysis B‐Environmental 108 (2011): 134–139.

[29] X. Zhang , K. Yue , R. Rao , et al., “Synthesis of acidic MIL‐125 from plastic waste: Significant contribution of N orbital for efficient photocatalytic degradation of chlorobenzene and toluene,” Applied Catalysis B‐Environmental 310 (2022): 121300.

[30] L. Qin , F. Sun , Z. Gong , et al., “Electrochemical NO 3– Reduction Catalyzed by Atomically Precise Ag 30 Pd 4 Bimetallic Nanocluster: Synergistic Catalysis or Tandem Catalysis?,” ACS Nano 17 (2023): 12747–12758. 37377221 10.1021/acsnano.3c03692

[31] X. Li , W. Zhang , W. Cui , et al., “Reactant activation and photocatalysis mechanisms on Bi‐metal@Bi_2_GeO_5_ with oxygen vacancies: A combined experimental and theoretical investigation,” Chemical Engineering Journal 370 (2019): 1366–1375.

[32] P. Chen , H. Liu , Y. Sun , et al., “Bi metal prevents the deactivation of oxygen vacancies in Bi_2_O_2_CO_3_ for stable and efficient photocatalytic NO abatement,” Applied Catalysis B‐Environmental Energy 264 (2020): 118545.

[33] R. Hailili , H. Ji , K. Wang , et al., “ZnO with controllable oxygen vacancies for photocatalytic nitrogen oxide removal,” ACS Catalysis 12 (2022): 10004–10017.

[34] Y. Jing , A. D. Fan , J. X. Guo , T. Shen , S. D. Yuan , and Y. H. Chu , “Synthesis of an ultrathin MnO_2_ nanosheet‐coated Bi_2_WO_6_ nanosheet as a heterojunction photocatalyst with enhanced photocatalytic activity,” Chemical Engineering Journal 429 (2022): 132193.

