# Supplementary material for: Boosting Bulk‐to‐Surface Electron Transfer in CeO2 via Oxygen Vacancy Channels for Ultrafast NO2 Sensing
Source: Adv Sci (Weinh). 2026 Jan 20;13(17):e23186. doi: 10.1002/advs.202523186 (PMC13042542; doi:10.1002/advs.202523186)
Supplement: Supplementary file 1 — Supporting File: advs73831‐sup‐0001‐SuppMat.doc. [file ADVS-13-e23186-s001.doc]

**Boosting Bulk-to-Surface Electron Transfer in CeO_2_ via Oxygen Vacancy Channels for Ultrafast NO_2_ Sensing**

Yucheng Ou^a^, Fuwen Wang^b^, Nana Xu^c^***, Haiyang Song^a^, Tao Liu^b^, Bing Wang^b^***, Ming Zhang^d^, Lei Liao^d^, Hui Xu^a^, Haijun Liu^a^***, Qingjiang Li^a^ , Wei Wang^a^***

*^a^College of Electronic Science and Technology, National University of Defense Technology, Changsha 410073, China*

*^b^Science and Technology on Advanced Ceramic Fiber and Composites Laboratory, College of Aerospace Science and Engineering,* *National University of Defense Technology, Changsha 410073, China*

*^c^School of Materials Science and Engineering, Hunan University of Science and Technology, Xiangtan 411201, China*

*^d^Changsha Semiconductor Technology and Application Innovation Research Institute, College of Semiconductors (College of Integrated Circuits), Hunan University, Changsha, China.*

**Contents**

**I. Experimental Section**

**II. Supplementary Figures**

**Figs. S1 to S20**

**Experimental Section**

**Methods**

**Chemicals and Materials.**

Cerous nitrate hexahydrate (Ce(NO_3_)_3_·6H_2_O, 99.95%) were purchased from Aladdin, sodium hydroxide (NaOH, ≧98%) were purchased from Aladdin. The water used in all experiments was ultrapure (18.2 Ω/cm).

**Synthesis of CeO_2_.** CeO_2_ was synthesized by hydrothermal method as well. 0.868 g Ce(NO_3_)_3_·6H_2_O and 9.6 g NaOH are dissolved in 35 mL and 5 ml of distilled water, respectively. As the clear solution formed, NaOH aqueous solution is slowly added to the Ce(NO_3_)_3_·6H_2_O with vigorous stirring. After stirring for about 30 mins, and mixture solution was subjected to hydrothermal treatment at 180 ^o^C for 24 h. The obtained powders were washed with water and ethanol for three cycles, dried in vacuum over a night, and further subjected to calcination at 550 ^o^C for 4 h.

**Synthesis of CeO_2_-200, CeO_2_-400 and CeO_2_-600.** CeO_2_-200, CeO_2_-400 and CeO_2_-600 are synthesized by the high temperature heat treatment at 200^o^C, 400^o^C and 600^o^C for 1.5 h under vacuum.

**Fabrication and testing of a gas sensor and experimental detail.**

5 mg of samples are mixed with 50 μL of deionized water to obtain the corresponding slurry. 5 μL of slurry was then dripped on an Pt interdigitated electrodes (10 mm × 5 mm × 0.25 mm, AURORA technologies, China) to form a resistance-type sensor. All the fabricated sensors were aged in air at 80 °C for 2 h. The gas-sensing performance of the fabricated sensors was evaluated using an intelligent gas-sensing analysis system (CGS-4TPs, Beijing Elite Tech Co., Ltd.). To produce test gases with the necessary concentrations, a dynamic gas and liquid distribution system (DGL-III, Beijing Elite Tech Co., Ltd, China) having three mass flow controllers was used. We use nitrogen as the carrier gas, and control the gas flow of oxygen and nitrogen to control the different oxygen concentrations in the reaction chamber. The interference gas is injected into the reaction chamber through a syringe. The room where the gas-sensitive analysis system is located is equipped with a constant temperature and humidity air conditioning system, and the indoor humidity can be artificially controlled by setting parameters. The response value (*S*_g_) is defined as the ratio of resistance in target (*R*_g_) to resistance in air gas (*R*_a_). The response time and recovery time are defined as the time for the sensor to reach 90% of the final signal.

**Characterization and measurements.** X-ray diffraction (XRD) patterns were recorded on a Bruker D8 X-ray powder diffractometer with Cu Kα radiation (λ = 1.5418 Å) at 30 kV and 10 mA with a scanning rate of 5° min^-1^ in the 2θ range of 10° ~ 80°. The HRTEM images were taken on a Talos F200i working at 200 kV and JEOL JEM-2100F field emission transmission electron microscopy with an accelerating voltage of 200 kV. The high-angle annular dark-field scanning transmission electron microscopy (HAADF-STEM) images and Xray energy dispersive spectroscopy (EDS) mapping was recorded on aberration-corrected TEM (FEI Titan Cubed Themis G2 300) at an accelerating voltage of 300 kV. Si (Li) EDS detector with a solid angle of 0.13 srad and Fiori number > 4000. 1.85 s/pixel and gather a 460 pixel map, so the time of EDS acquisition will cost 851 seconds. The In situ Fourier-transform infrared (In situ FT-IR) spectra were recorded on a Bruker Vertex 70 FTIR spectrometer equipped with in situ reaction chamber. X-band electron paramagnetic resonance (EPR) measurement was performed at room temperature using a JEOL FA-200 EPR spectrometer. The X-ray photoelectron spectroscopy (Axis Supra) measurements were operated with Al Ka radiation (1486.6 eV). Binding energies (BE) were calibrated by setting the measured BE of C 1s to 284.8 eV. In situ DRIFTS spectra were measured on a Nicolet-6700 FTIR spectrometer. The N_2_ adsorption-desorption isotherms were measured using a BELSORP-max-II to estimate specific surface area and pore size distribution by the Brunauer-Emmett-Teller (BET) and Barrett–Joyner–Halenda (BJH) methods. The process Raman system employed in this study was HORIBA. X-ray absorption fine spectra (XAFS) measurements were measured on the B11 station in Shanghai Synchrotron Radiation Facility (SSRF). **X-ray absorption data analysis**

The obtained XAFS data was processed in Athena (version 0.9.26) for background, pre-edge line and post-edge line calibrations. Then Fourier transformed fitting was carried out in Artemis (version 0.9.26). The k2 weighting, k-range of 2-10 Å^-1^ and R range of 1-3 Å were used for the fitting of CeO_2_ and Sample.

**NAP-XPS Measurement Conditions**

Pressure: The experiments were conducted in a near-ambient pressure cell. The total pressure was maintained at 0.2 mbar for the O_2_ environment and 0.4 mbar for the O_2_ + NO_2_ mixture environment.

Temperature: All NAP-XPS measurements were performed at room temperature (~25 °C).

Gas Environment: The gas atmosphere during measurements consisted of pure O_2_ (for the O_2_ condition) and a mixture of O_2_ and NO_2_ (for the O_2_+NO_2_ condition), as relevant to the specific experiment. The composition is now explicitly stated in the revised text.

Calibration: The binding energy scale for all spectra was calibrated using the adventitious carbon (C 1s) peak, set at 284.6 eV.

**DFT calculations.**

DFT calculations are carried out using the VASP code. The projector augmented-wave (PAW) method and Perdew-Burke-Ernzerhof generalized gradient approximation (GGA-PBE) ae used for the exchange correlation functionals. The energy cutoff of 400 eV is used. The molecular dynamics simulations are carried out in the canonical ensemble (NVT) with the Nose-Hoover thermostat to generate amorphous models. The time step is set to 1 fs. All initial amorphous structures are thermally equilibrated at ambient temperature for 25 ps. The energy and force on each ion are reduced below 10^-5^ eV/atom and 0.01 eV/A, respectively, and only the Γ point was sampled from the Brillouin zone. The amorphous models were obtained by using the heat up process: from 0 to 900 K within 25 ps to obtain the structural snapshots and total energy distributions at the corresponding temperatures. The effect of core electrons on the density of valence electrons was described using the projector augmented wave method. The kinetic energy cutoff for the plane waves was set to 450 eV for all the calculations. To consider the open-shell d-electrons, GGA+U schemes were implemented, employing effective U values of 5.0 for Ce. The convergence tolerance of energy and force on each atom during structure relaxation were less than 10^−5^ eV and 0.03 eV/Å, respectively. The equipotential surfaces is set at 0.005 e/Å^3^. A set of Monkhorst–Pack mesh K points of 2×2×1 and 4×4×1 is used to sample the Brillouin zone for geometry optimization and electronic structural calculations. A vacuum distance of 15 Å was set to ensure sufficient vacuum and avoid interactions between two periods. Further calculations were carried out to determine the thermal and zero-point energy (ZPE) corrections at the Γ point of various intermediates adsorbed on the surface. The VASPKIT code was used for postprocessing computational data obtained from VASP.

The adsorption energy can be evaluated by ΔG_ads_, which is defined as

ΔE_ads_ = E_(System+ gas)_ − E_(System)_ −1/2 E _ads_,

in which E_(System+ gas)_ and E_(System)_ are the energies of all research systems with and without gas adsorption, respectively. Eads represents the energy of adsorbed intermediates.

**II. Supplementary Figure**


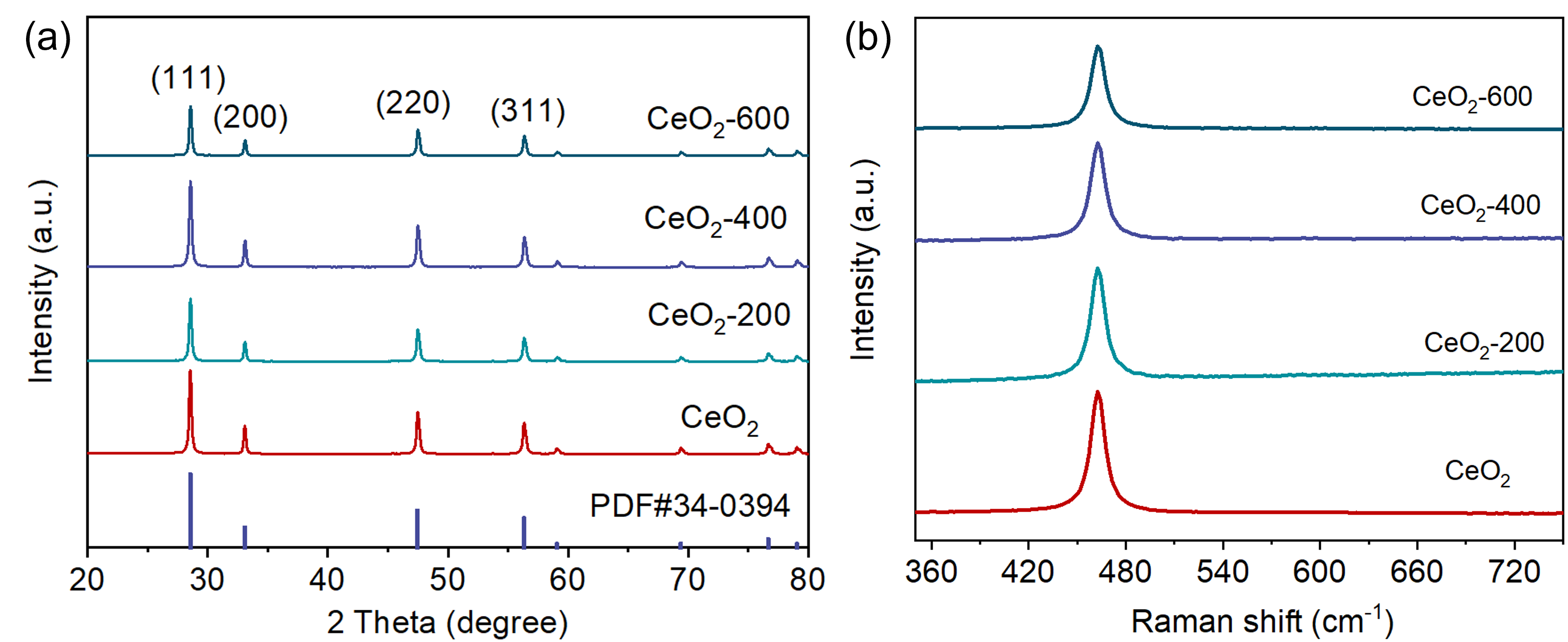


**Fig. S1.** a XRD pattern of CeO_2_, CeO_2_-200, CeO_2_-400 and CeO_2_-600. b Raman spectra of CeO_2_, CeO_2_-200, CeO_2_-400 and CeO_2_-600.


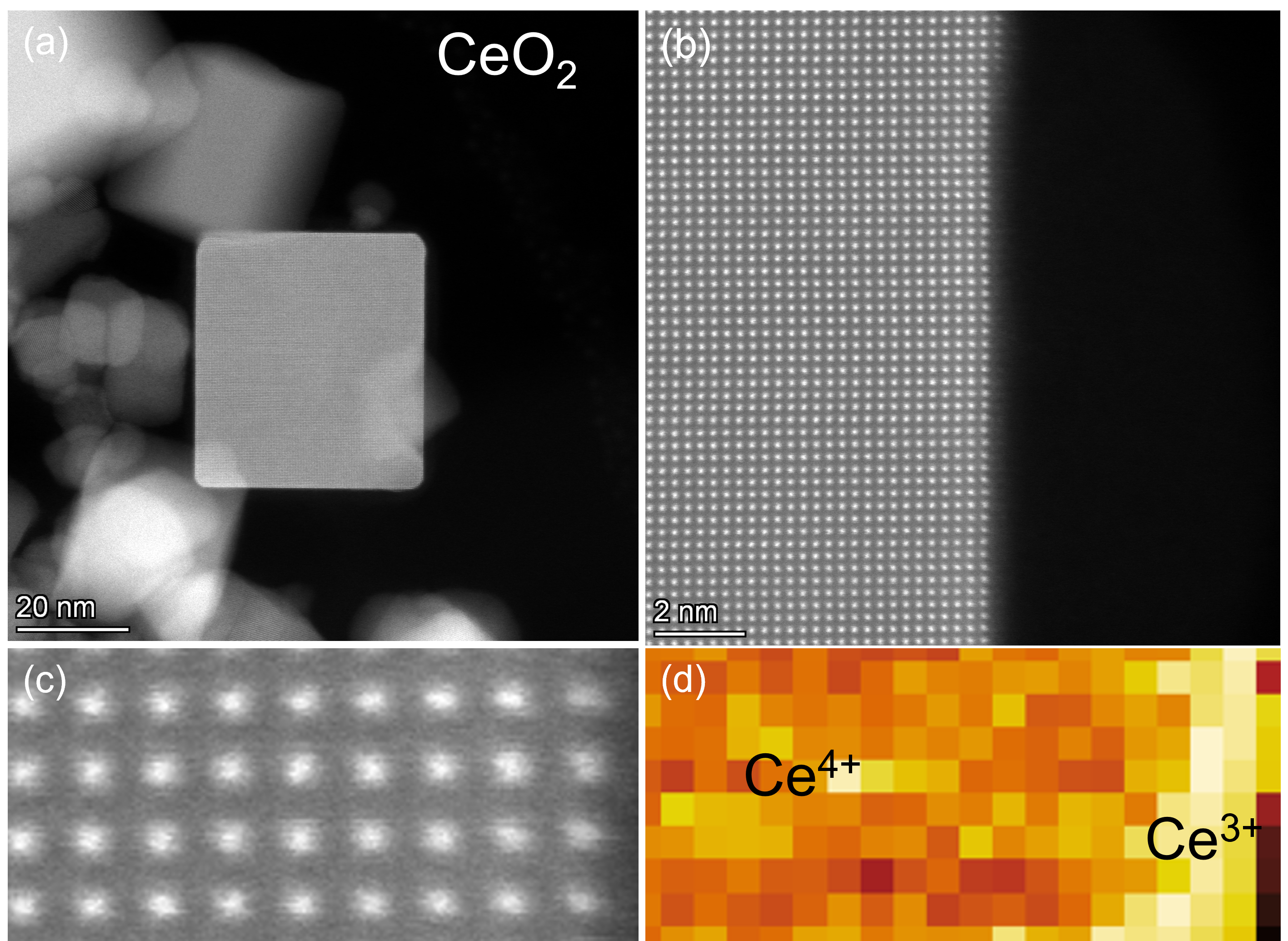


**Fig. S2.** AC-TEM of CeO_2_.


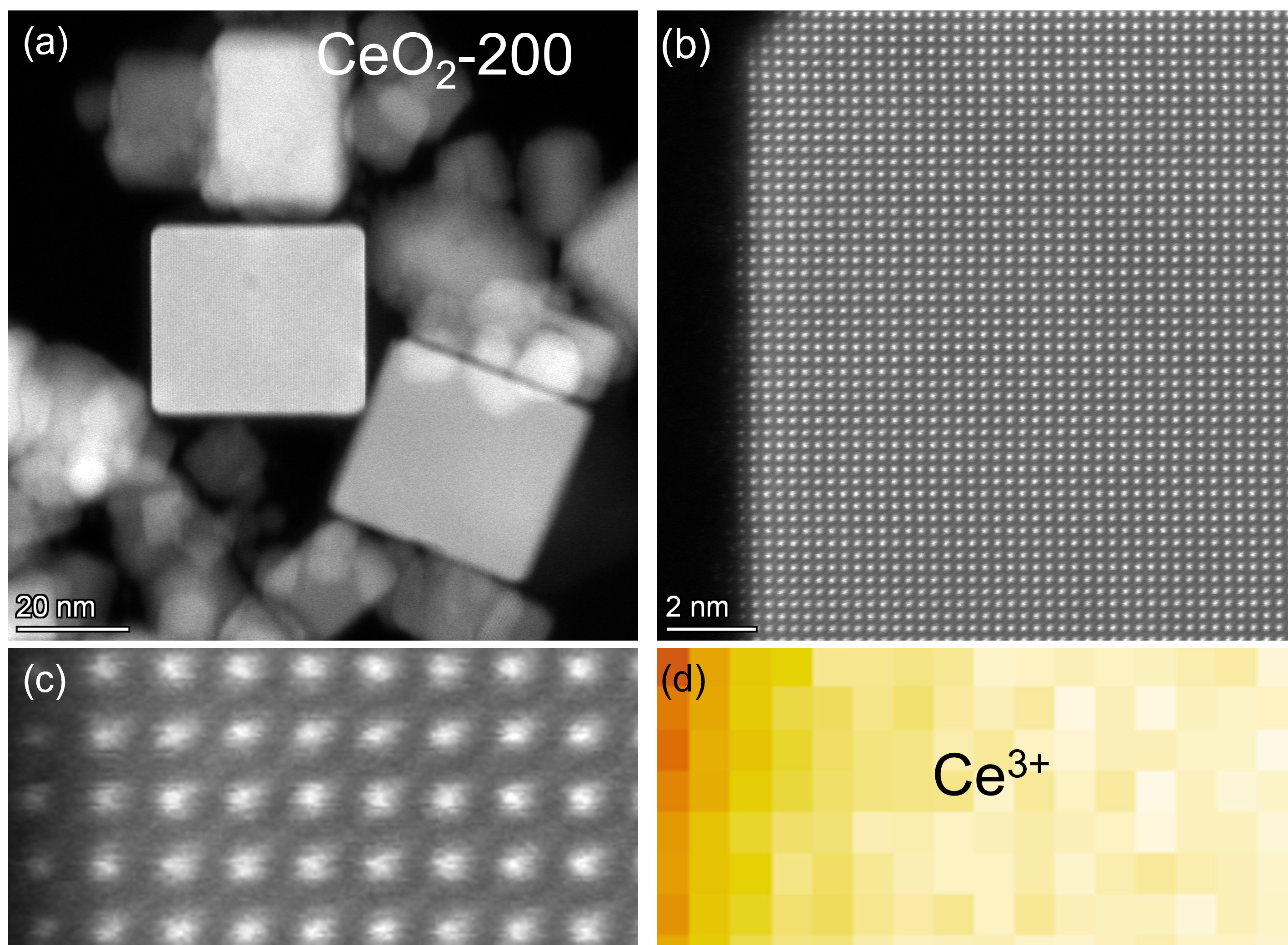


**Fig. S3.** AC-TEM of CeO_2_-200.


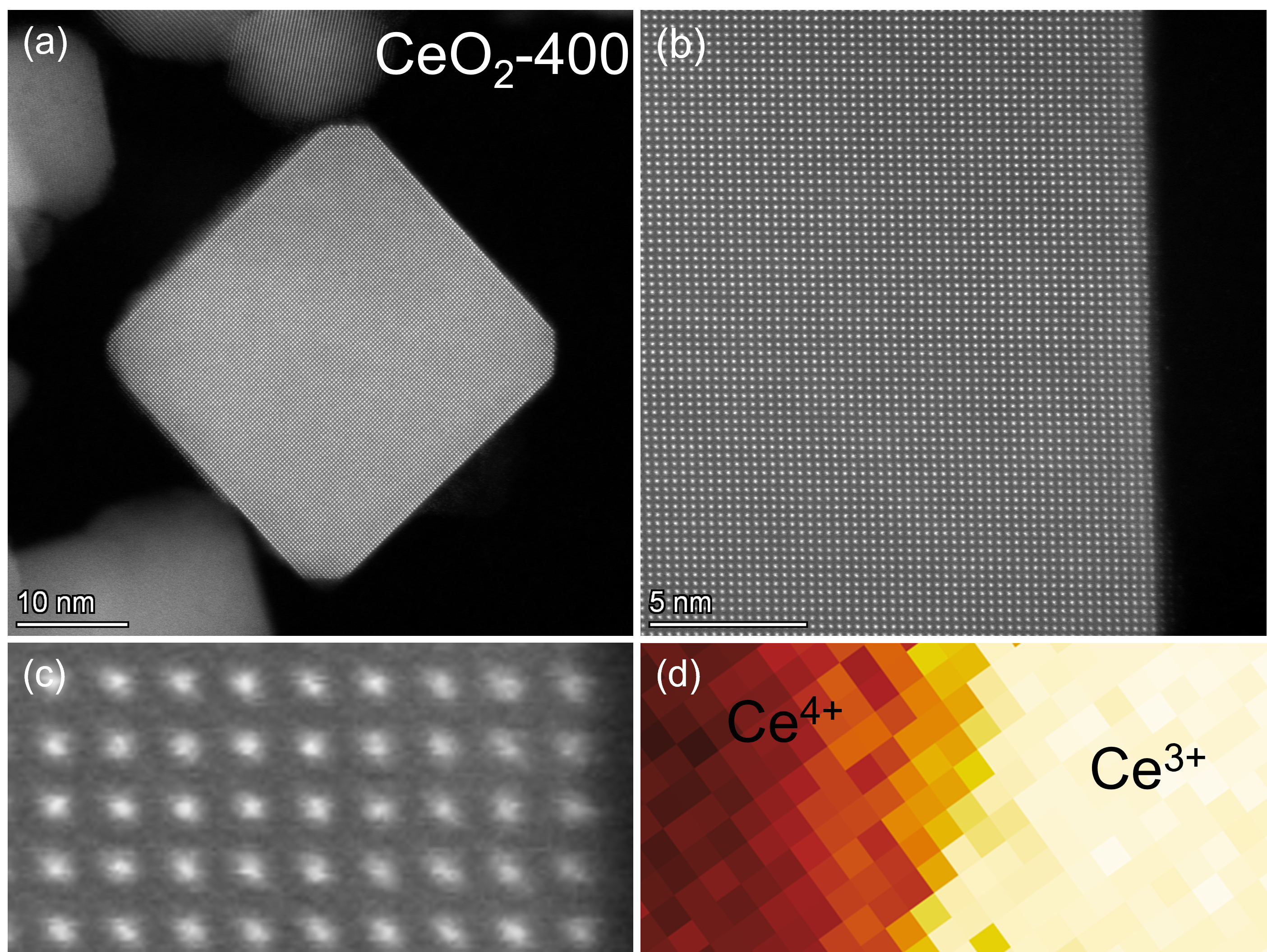


**Fig. S4.** AC-TEM of CeO_2_-400.


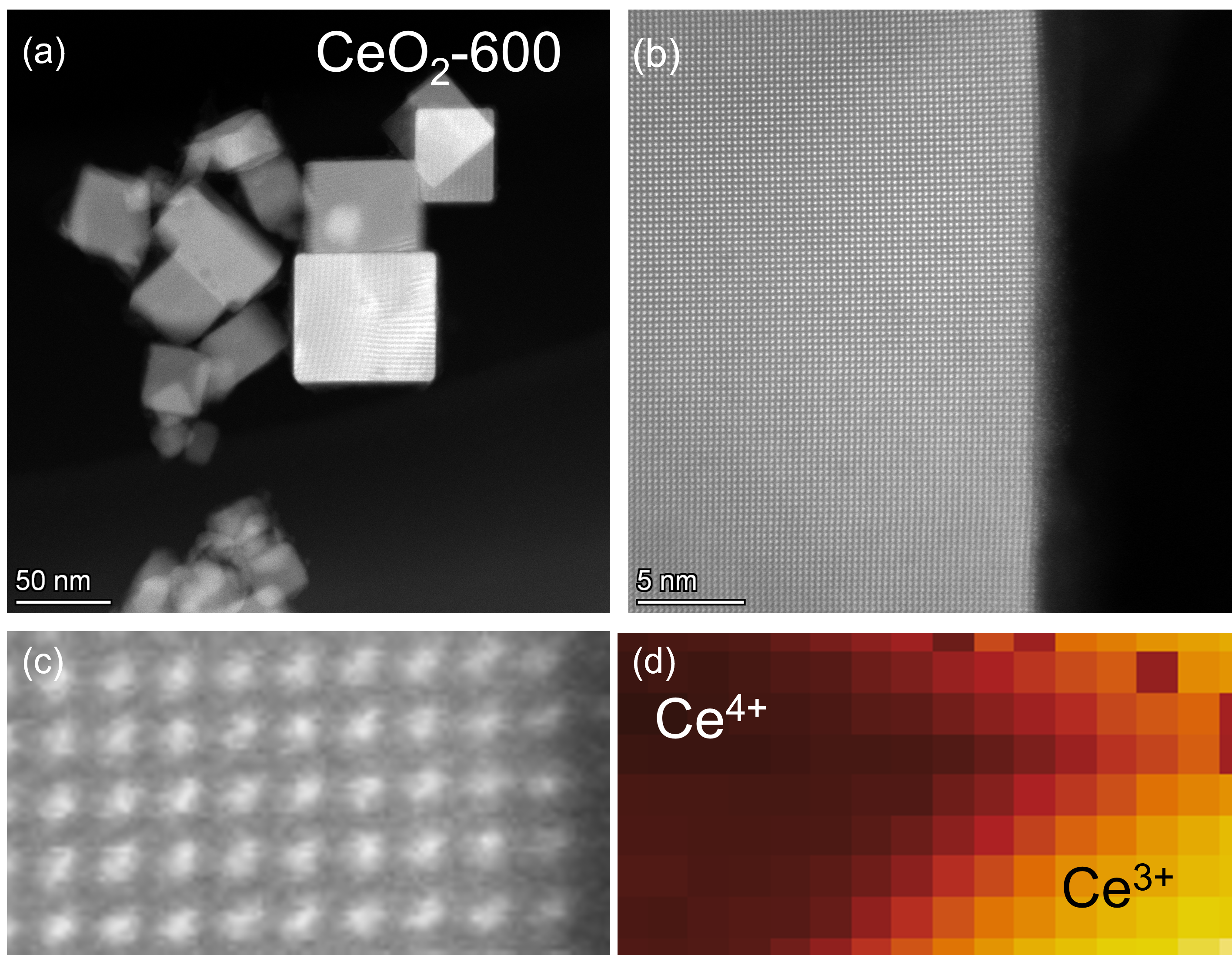


**Fig. S5.** AC-TEM of CeO_2_-600.


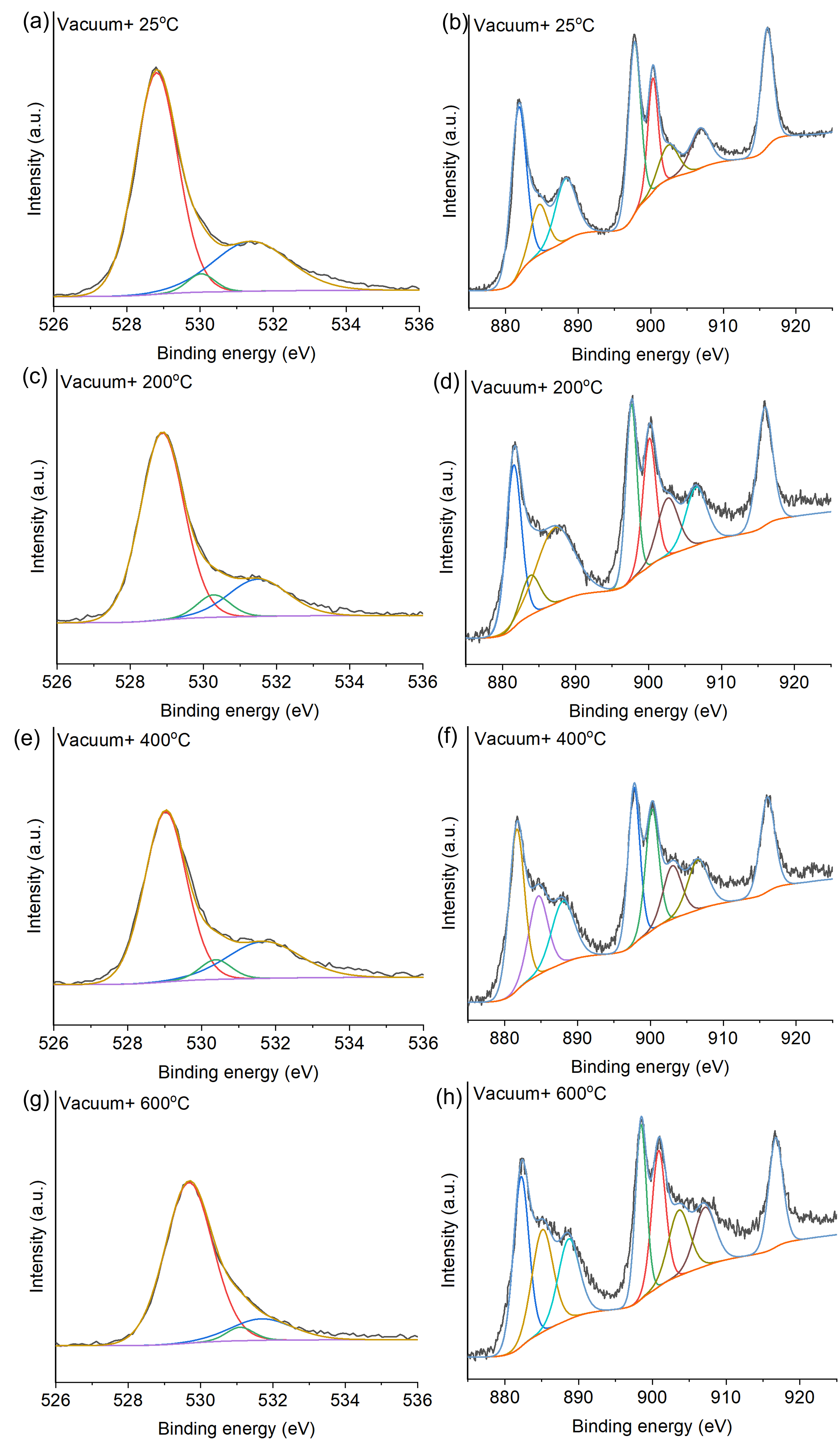


**Fig. S6.** *In-situ* NAP-XPS analysis of CeO_2_.


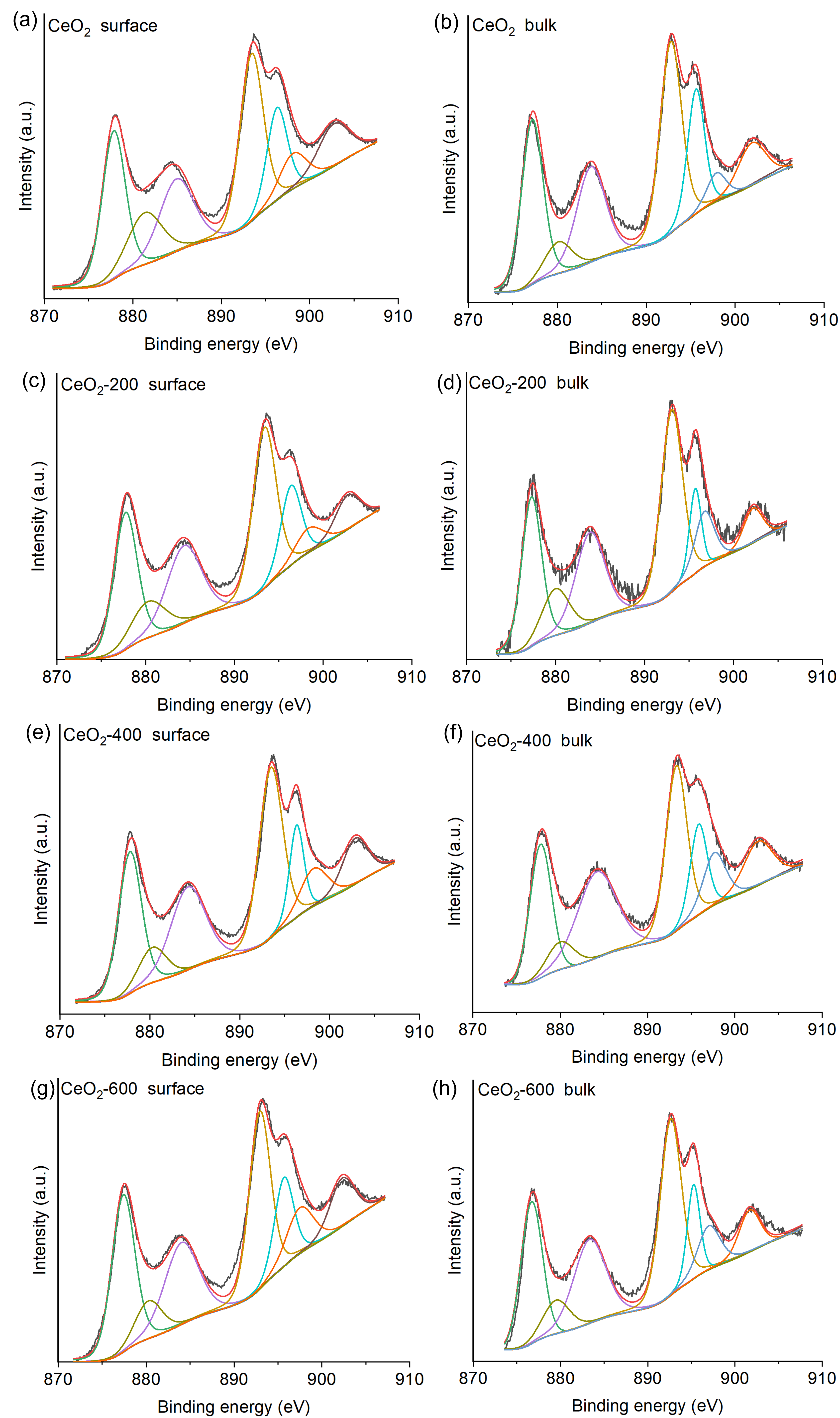


**Fig. S7.** HAXPES analysis of CeO_2_, CeO_2_-200, CeO_2_-400 and CeO_2_-600.


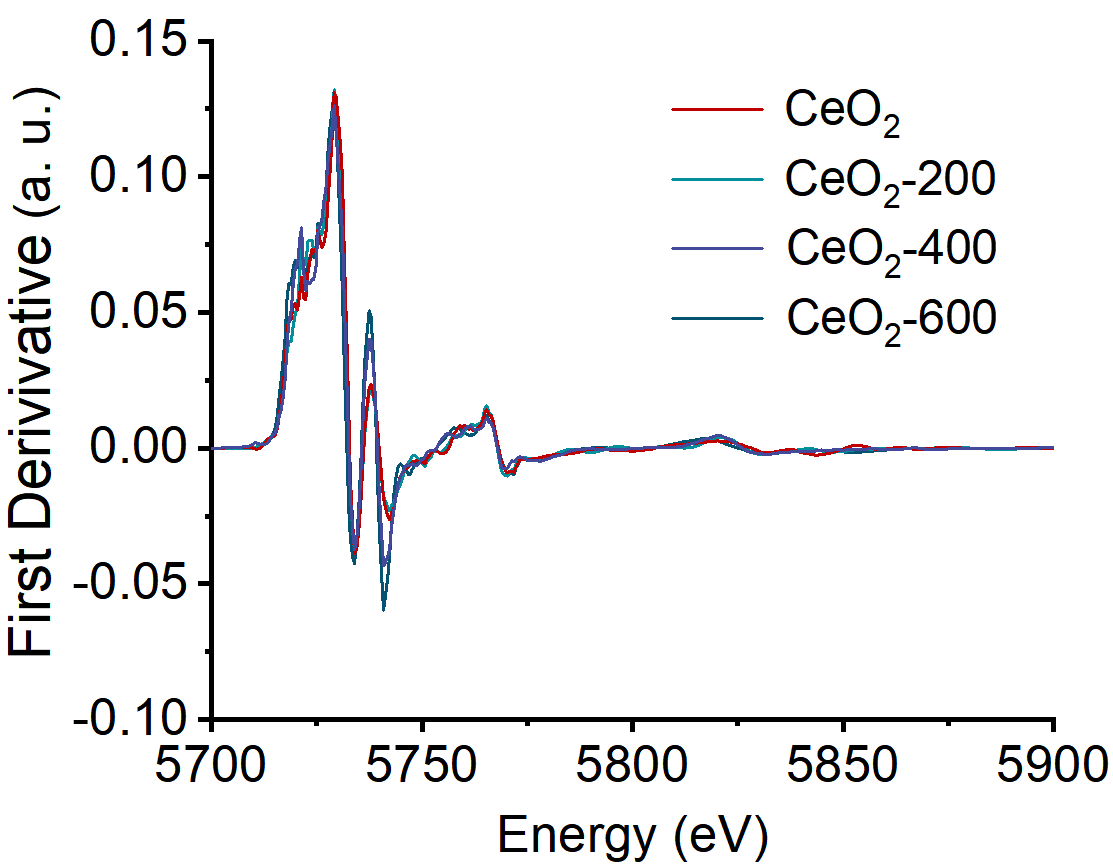


**Fig. S8.** Ce L3-edge XANES spectra


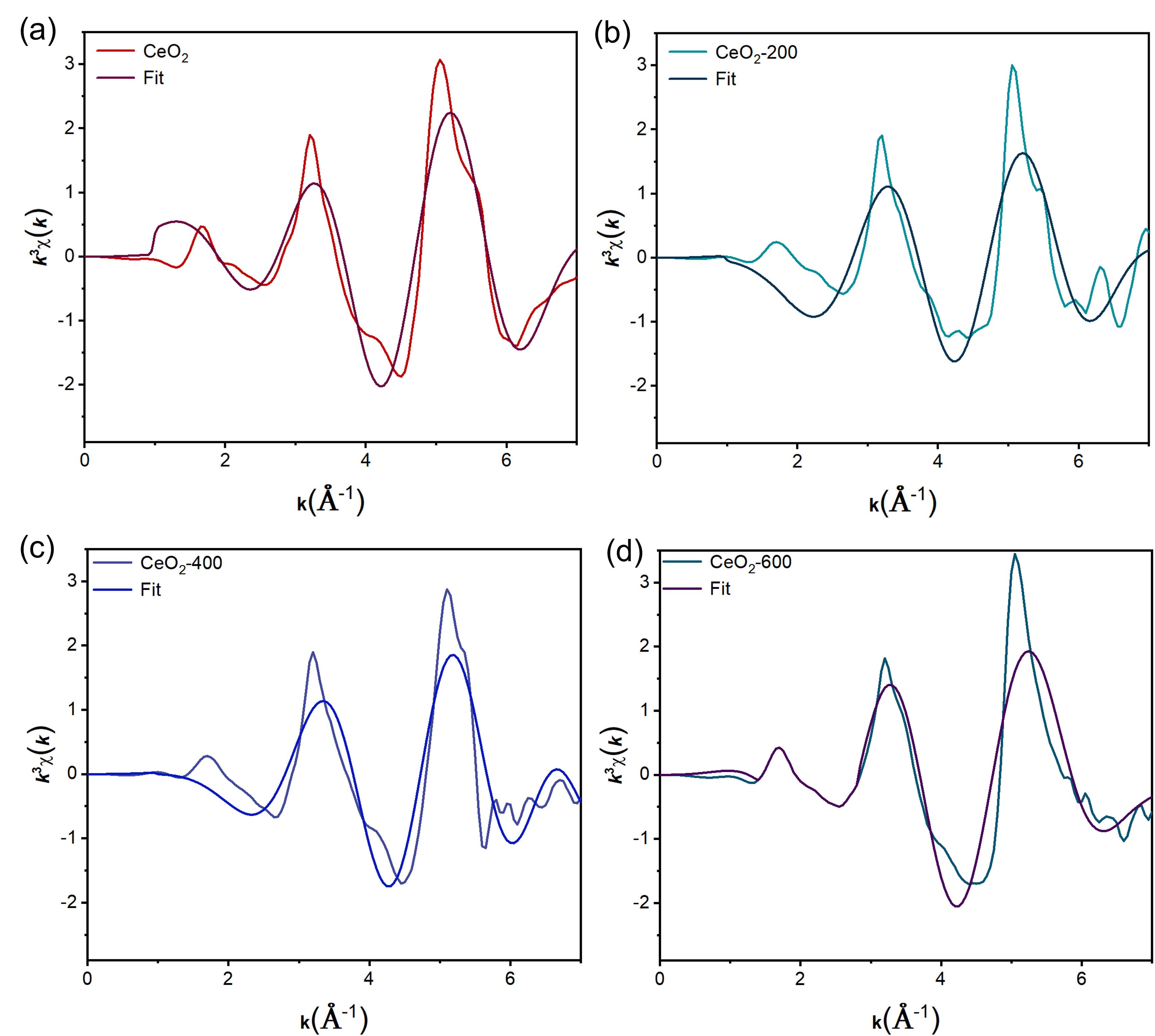


**Fig. S9.** Experimental and fitting results of Fourier transformed extended X-ray absorption fine structure (EXAFS) spectra of CeO_2_, CeO_2_-200, CeO_2_-400 and CeO_2_-600.


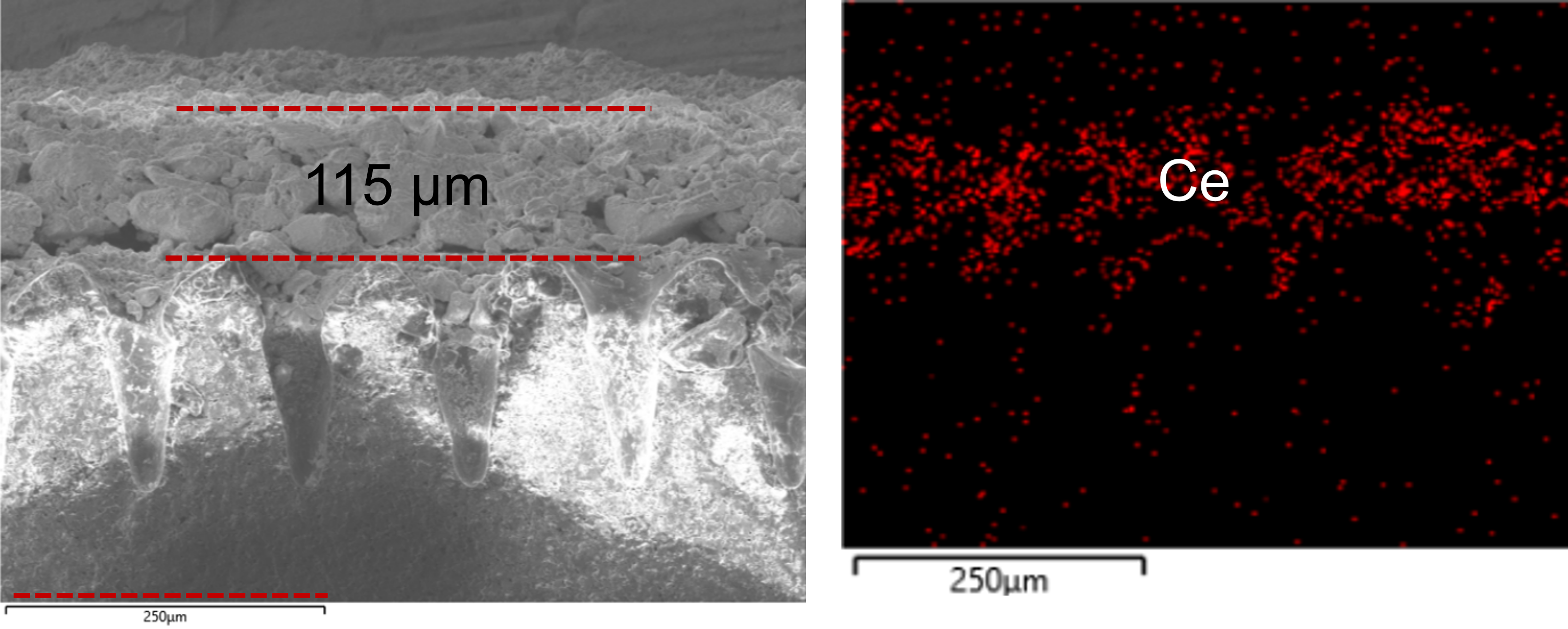


**Fig. S10.** The thickness of sensing layer.


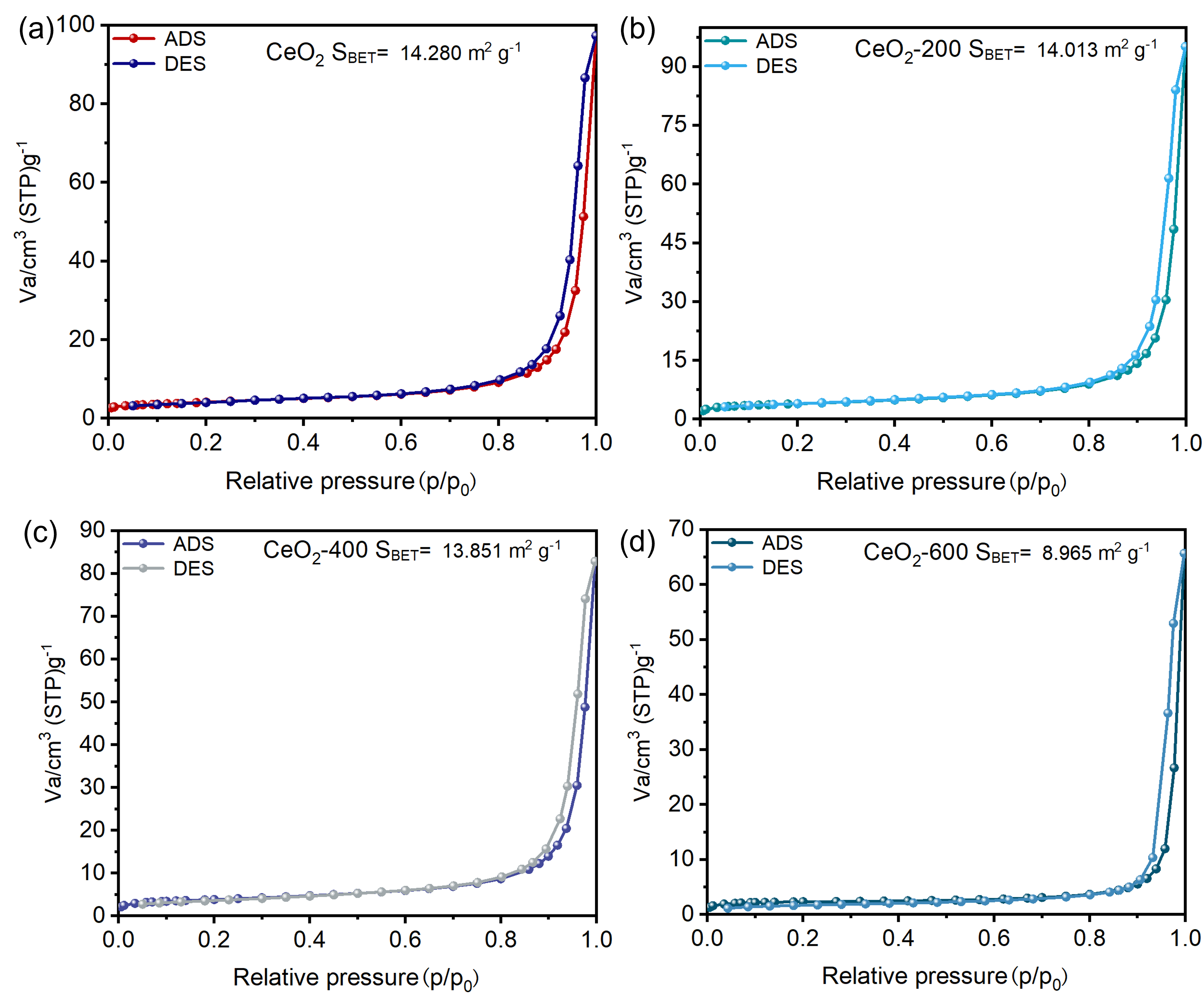


**Fig. S11.** BET of CeO_2_, CeO_2_-200, CeO_2_-400 and CeO_2_-600.


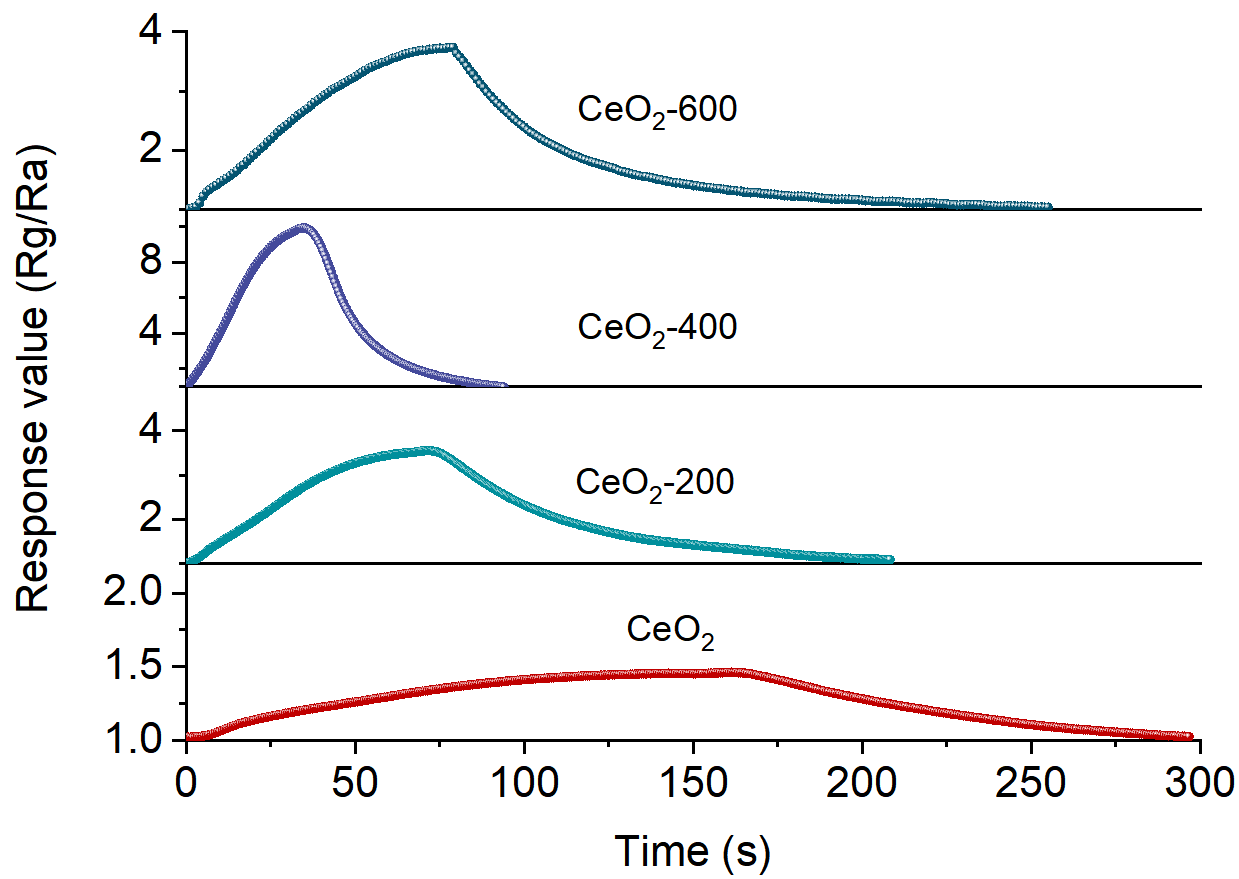


**Fig. S12.** Response-recovery time of CeO_2_, CeO_2_-200, CeO_2_-400 and CeO_2_-600.


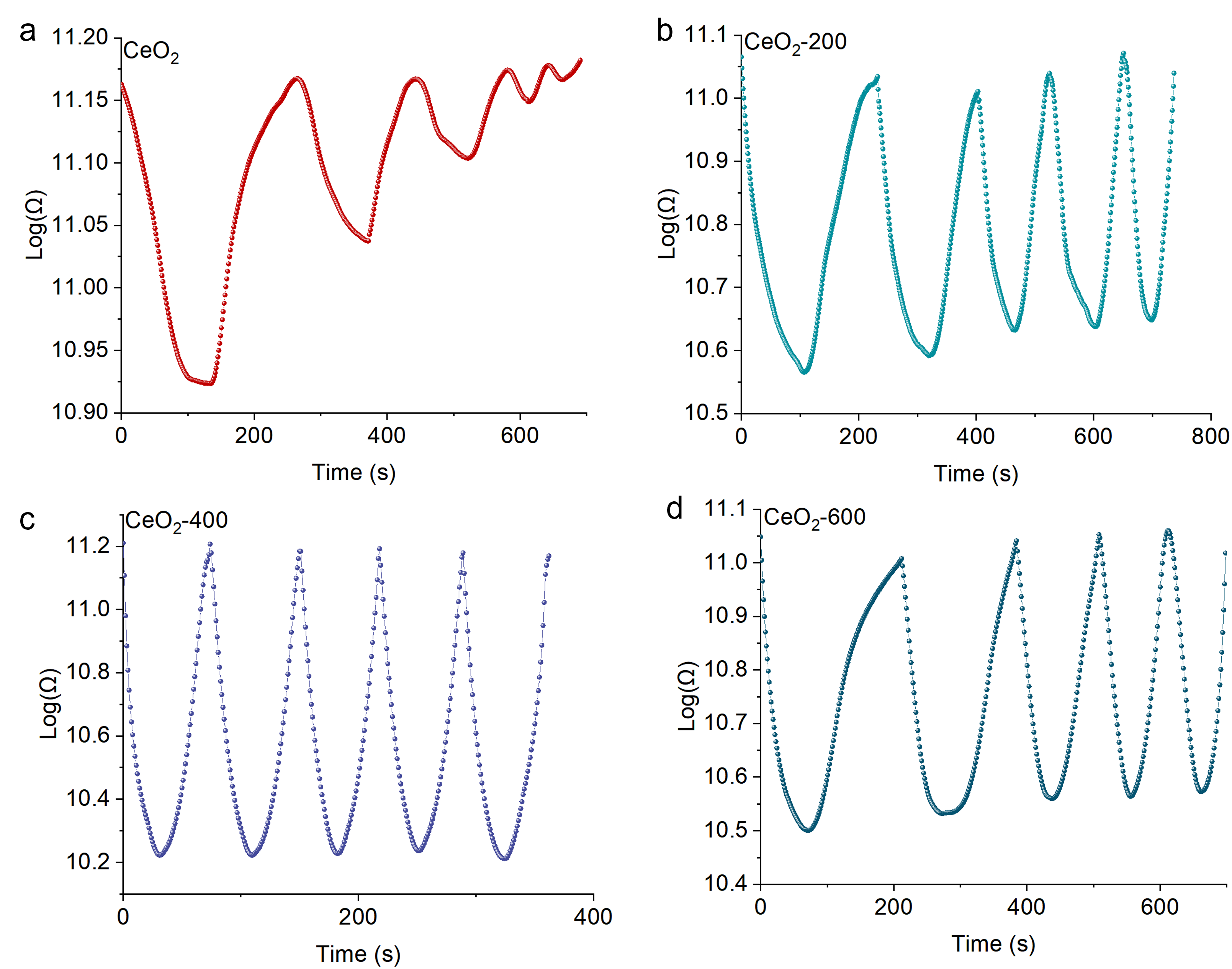


Fig. S13. Full resistance-versus-time trace for five consecutive sensing cycles


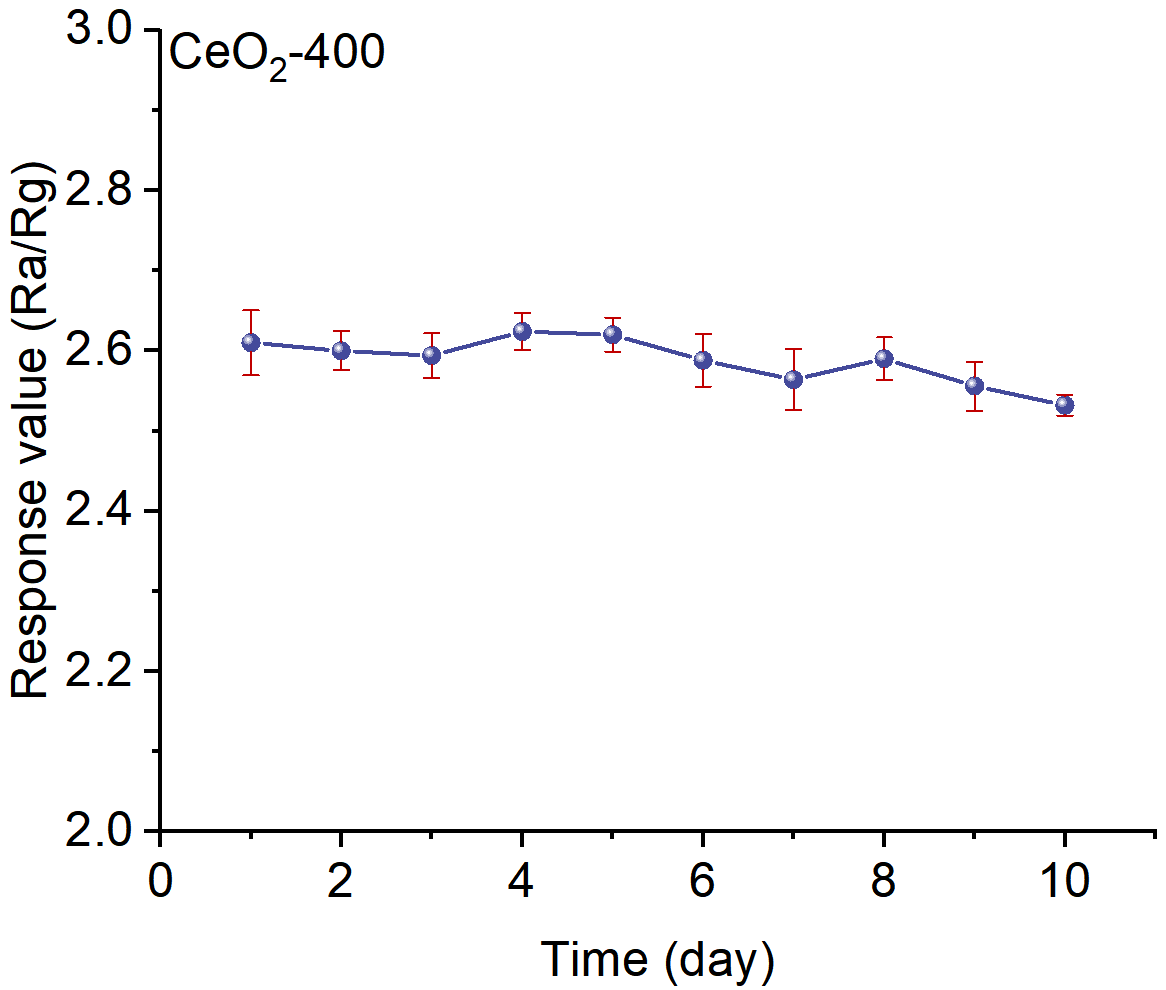


Fig. S14. Long-term stability test


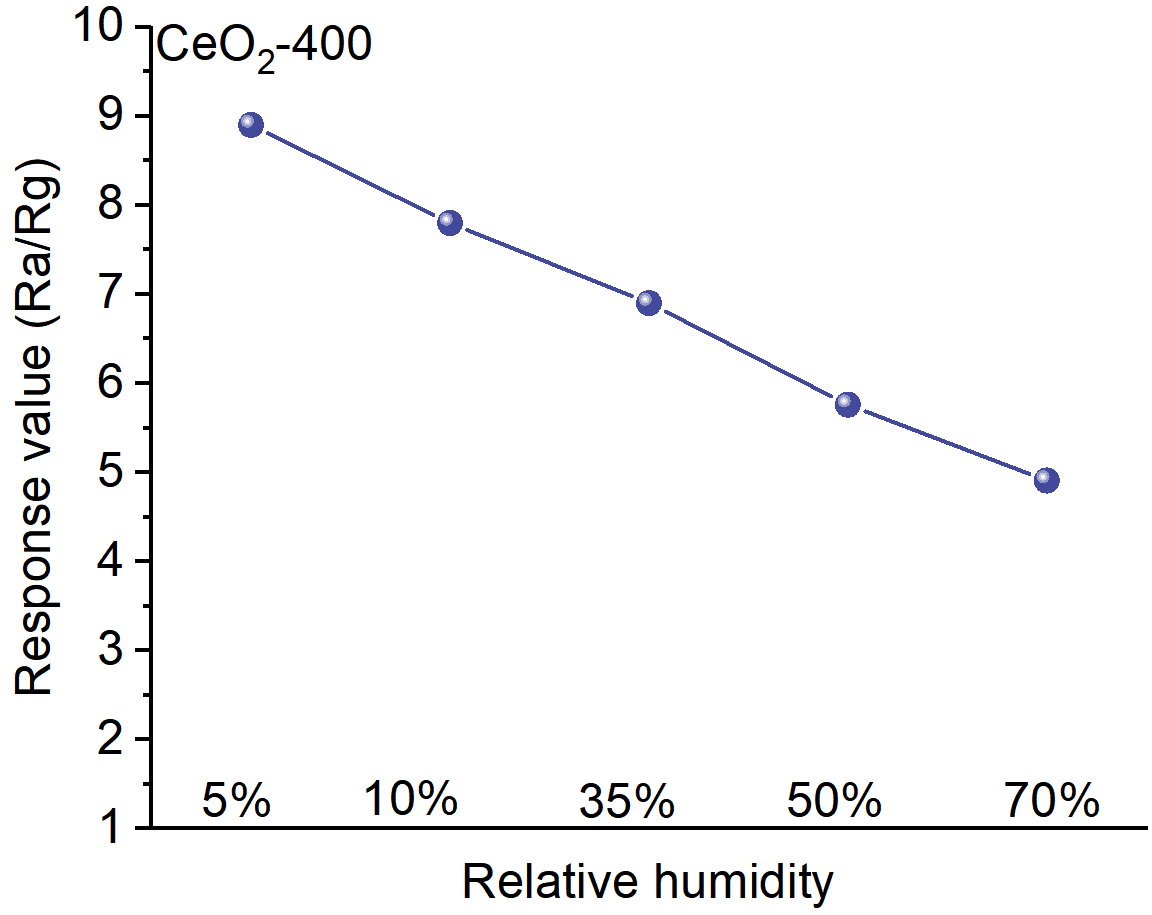


Fig. S15. Response value of CeO_2_-400 under different relative humidity


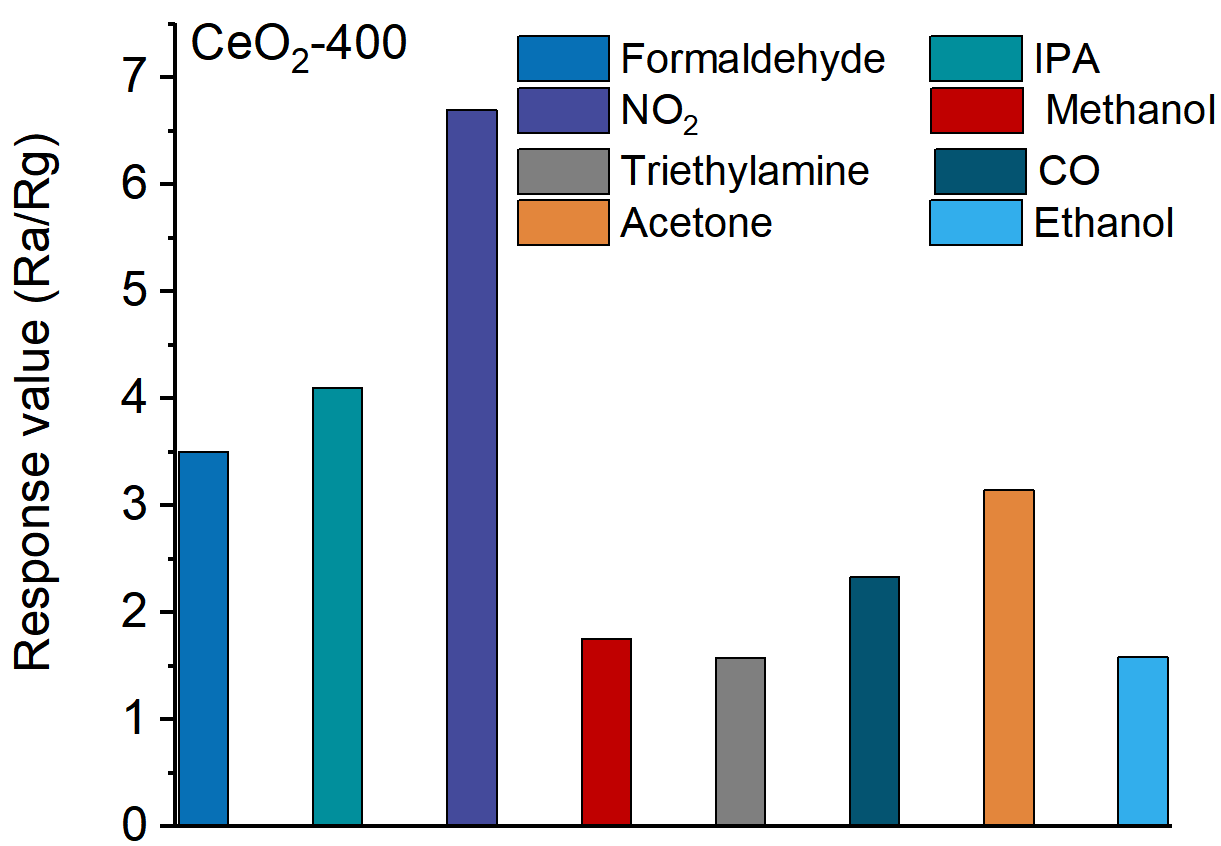


Fig. S16. Gas selectivity tests of CeO_2_-400


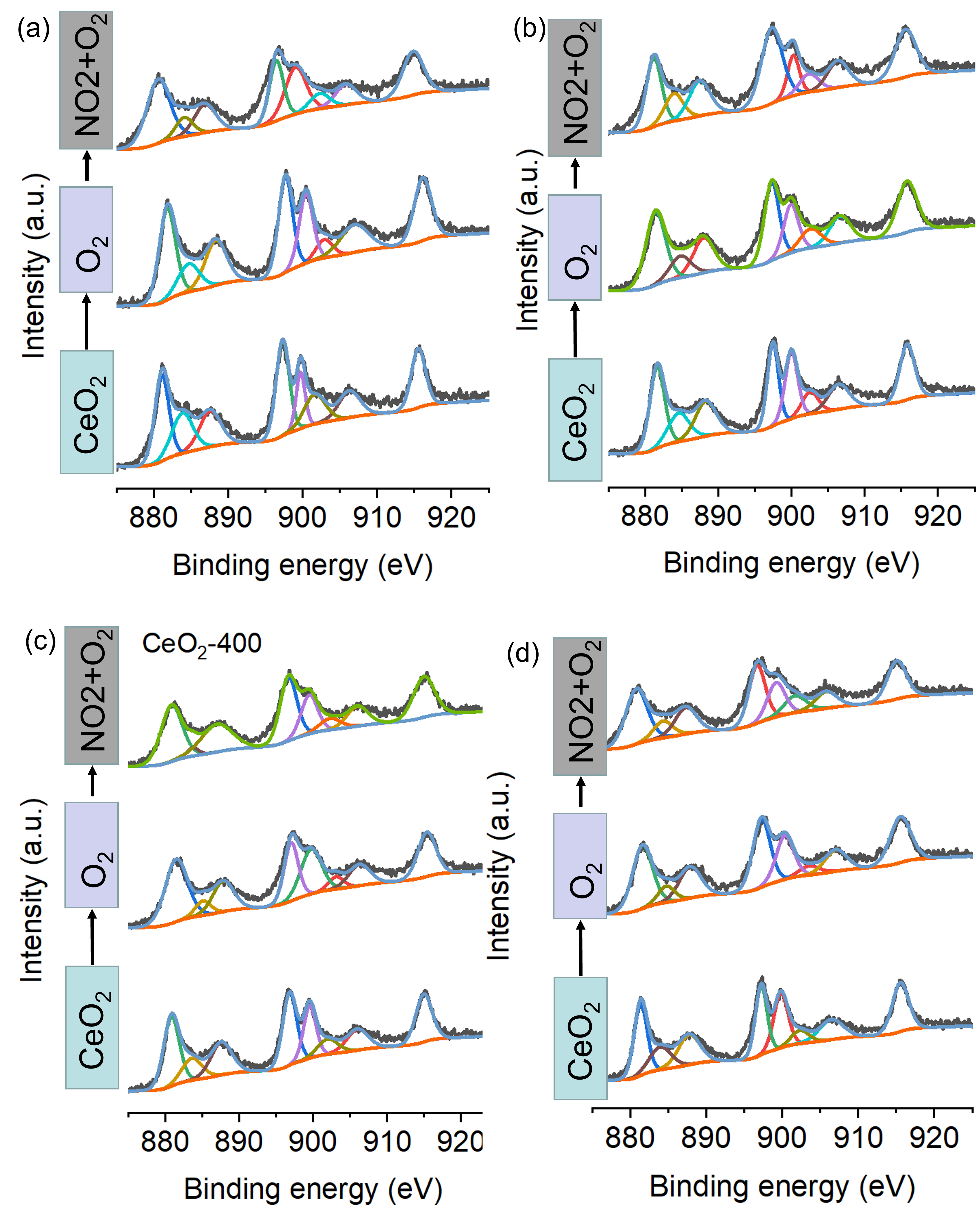


**Fig. S17.** NAP-XPS analysis of CeO_2_, CeO_2_-200, CeO_2_-400 and CeO_2_-600.


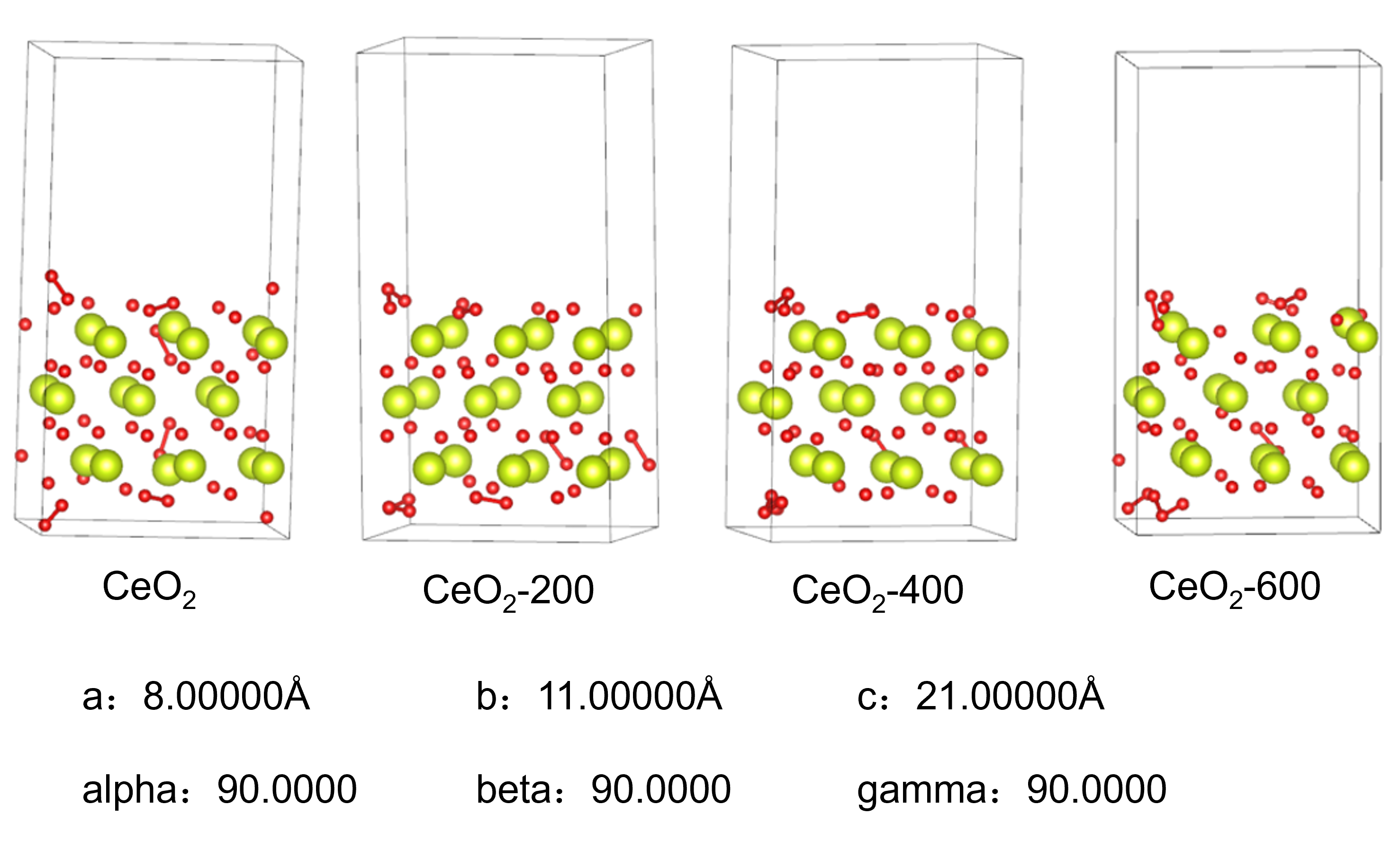


**Fig. S18.** Detailed information of DFT.


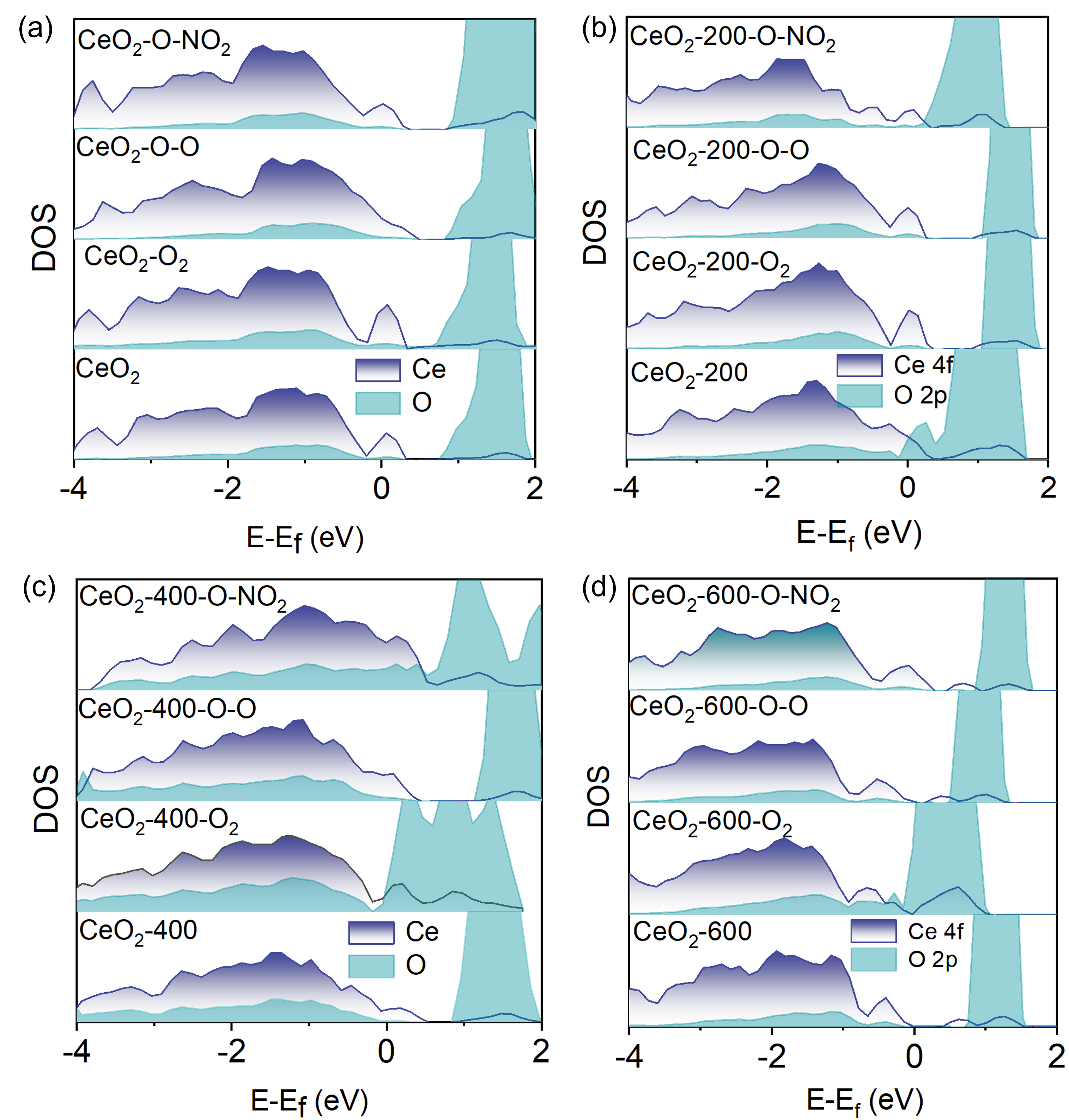


**Fig. S19.** DOS analysis of CeO_2_, CeO_2_-200，CeO_2_-400 and CeO_2_-600.


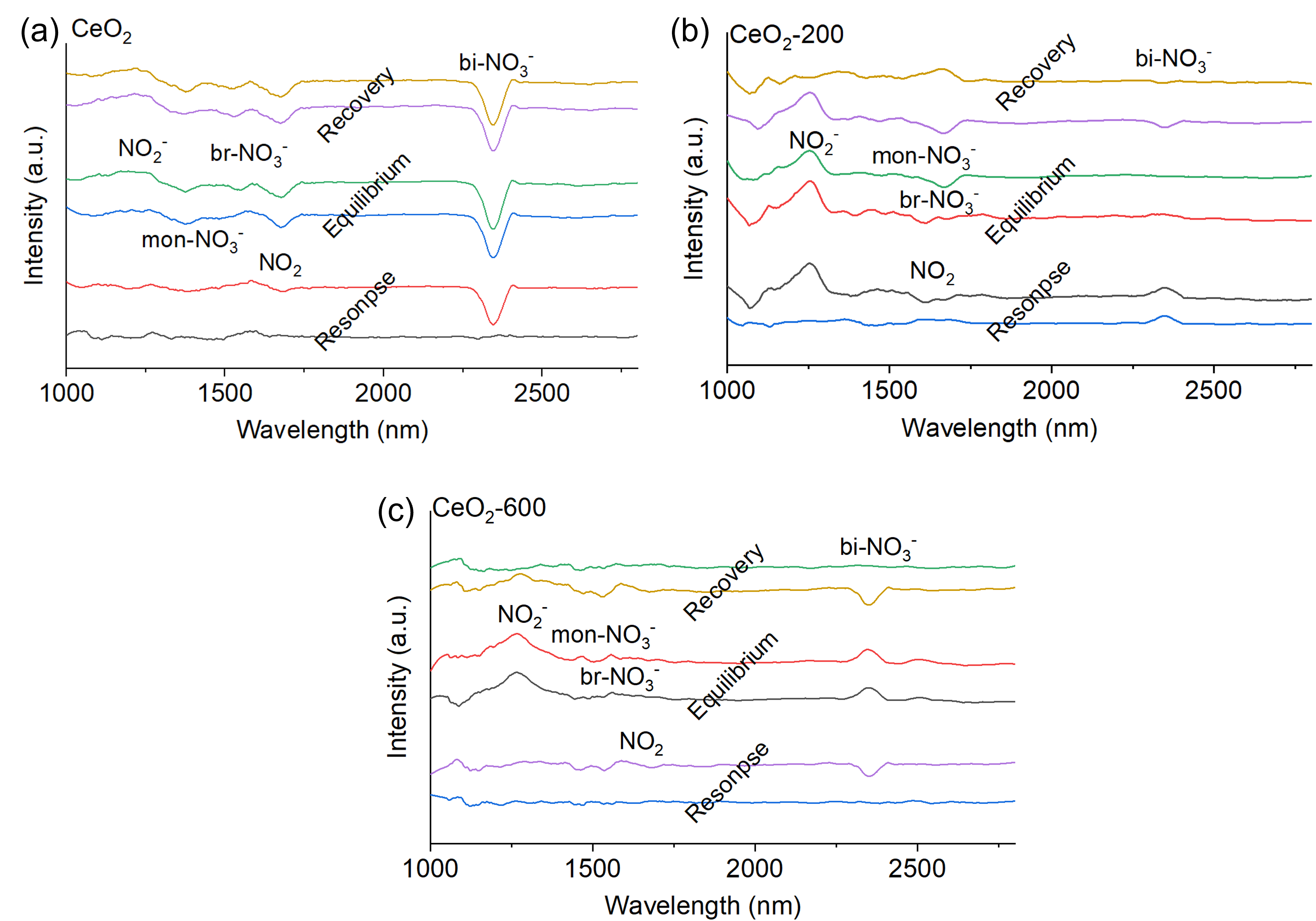


**Fig. S20.** *In-Situ* FTIR analysis of CeO_2_, CeO_2_-200 and CeO_2_-600.
